# Pre-existing cell subpopulations in primary prostate cancer tumors display surface fingerprints of docetaxel-resistant cells

**DOI:** 10.1007/s13402-024-00982-2

**Published:** 2024-08-20

**Authors:** Stanislav Drápela, Barbora Kvokačková, Eva Slabáková, Anna Kotrbová, Kristína Gömöryová, Radek Fedr, Daniela Kurfürstová, Martin Eliáš, Vladimír Študent jr, Frederika Lenčéšová, Ganji Sri Ranjani, Vendula Pospíchalová, Vítězslav Bryja, Wytske M. van Weerden, Martin Puhr, Zoran Culig, Jan Bouchal, Karel Souček

**Affiliations:** 1 Department of Cytokinetics, Institute of Biophysics of the Czech Academy of Sciences, Královopolská 135, Brno, 612 00 Czech Republic; 2 International Clinical Research Center, St. Anne’s University Hospital in Brno, Brno, 602 00 Czech Republic; 3 Department of Experimental Biology, Faculty of Science, Masaryk University, Brno, 625 00 Czech Republic; 4 Department of Clinical and Molecular Pathology, Institute of Molecular and Translational Medicine, Faculty of Medicine and Dentistry, Palacky University and University Hospital, Olomouc, 779 00 Czech Republic; 5 Department of Urology, University Hospital Olomouc, Olomouc, 779 00 Czech Republic; 6 Central European Institute of Technology, Masaryk University, 625 00 Brno, Czech Republic; 7 Department of Urology, Erasmus MC Cancer Institute, Erasmus University Medical Center, Wytemaweg 80, Rotterdam, 3015 CN The Netherlands; 8 Department of Urology, Experimental Urology, Medical University of Innsbruck, Anich Strasse 35, Innsbruck, A-6020 Austria; 9 Present Address: Department of Molecular Oncology, H. Lee Moffitt Cancer Center & Research Institute, FL 33612 Tampa, USA; 10 Present Address: Department of Experimental Biology, Faculty of Science, Masaryk University, Brno, 625 00 Czech Republic

**Keywords:** Prostate cancer, Docetaxel resistance, Intratumoral heterogeneity, Plasticity, CD95/Fas, SSEA-4

## Abstract

**Purpose:**

Docetaxel resistance is a significant obstacle in the treatment of prostate cancer (PCa), resulting in unfavorable patient prognoses. Intratumoral heterogeneity, often associated with epithelial-to-mesenchymal transition (EMT), has previously emerged as a phenomenon that facilitates adaptation to various stimuli, thus promoting cancer cell diversity and eventually resistance to chemotherapy, including docetaxel. Hence, understanding intratumoral heterogeneity is essential for better patient prognosis and the development of personalized treatment strategies.

**Methods:**

To address this, we employed a high-throughput single-cell flow cytometry approach to identify a specific surface fingerprint associated with docetaxel-resistance in PCa cells and complemented it with proteomic analysis of extracellular vesicles. We further validated selected antigens using docetaxel-resistant patient-derived xenografts in vivo and probed primary PCa specimens to interrogate of their surface fingerprint.

**Results:**

Our approaches revealed a 6-molecule surface fingerprint linked to docetaxel resistance in primary PCa specimens. We observed consistent overexpression of CD95 (FAS/APO-1), and SSEA-4 surface antigens in both in vitro and in vivo docetaxel-resistant models, which was also observed in a cell subpopulation of primary PCa tumors exhibiting EMT features. Furthermore, CD95, along with the essential enzymes involved in SSEA-4 synthesis, ST3GAL1, and ST3GAL2, displayed a significant increase in patients with PCa undergoing docetaxel-based therapy, correlating with poor survival outcomes.

**Conclusion:**

In summary, we demonstrate that the identified 6-molecule surface fingerprint associated with docetaxel resistance pre-exists in a subpopulation of primary PCa tumors before docetaxel treatment. Thus, this fingerprint warrants further validation as a promising predictive tool for docetaxel resistance in PCa patients prior to therapy initiation.

**Supplementary Information:**

The online version contains supplementary material available at 10.1007/s13402-024-00982-2.

## Introduction

Docetaxel resistance presents a substantial hurdle in prostate cancer (PCa) therapy ultimately resulting in disease relapse and death [1]. The resistance to docetaxel can typically emanate from two different evolutionary pathways, either *de novo* from drug-tolerant “persister” cells surviving docetaxel therapy or as a result of pre-existing intratumoral heterogeneity [2, 3]. The latter, clinically considered a *fait accompli*, posits that rare resistant clones emerge in the tumor mass before treatment and cause relapse after initial therapy intervention due to clonal selection and expansion [4, 5]. Failure to understand the extent of intratumoral heterogeneity and clonality in the context of therapy resistance remains one of the main bottlenecks of current PCa translation research [6–8]. Intratumoral heterogeneity is shaped to a significant extent by lineage plasticity, defined as a physiological process and the ability of a cell to reversibly or irreversibly modify its identity that differs in their original competence [9, 10]. In PCa cells, such context-dependent reprogramming of one committed phenotype is closely related to the formation of overt metastasis, acquisition of an invasive phenotype and development of therapy resistance often accompanied by epithelial-to-mesenchymal transition (EMT) [11–13]. Furthermore, intratumoral heterogeneity may be linked to docetaxel resistance in PCa through numerous other mechanisms, such as gene expression fluctuations, cell-cell communication dysfunction, increased drug efflux, and aberrations in the epigenetic landscape. Although previous studies have utilized high-throughput techniques such as single-cell RNAseq, proteome analysis, or ATACseq to understand intratumoral heterogeneity in docetaxel-resistant PCa, translation to clinical applications remains rather complicated [14–16].

Our study aimed to identify biomarkers to predict docetaxel resistance in PCa before the initiation of therapy. Here, we determined a 6-molecule surface fingerprint that reflects the docetaxel-resistant phenotype in various in vitro PCa models. As a part of this fingerprint, surface antigens CD95 and SSEA-4 were concordantly upregulated in both in vitro and in vivo docetaxel-resistant PCa models and in some probed clinical specimens displaying EMT features. CD95 and the enzymes responsible for SSEA-4 synthesis displayed significantly elevated levels in post-docetaxel-based therapy patients and were correlated with poor survival probability, highlighting their potential as reliable biomarkers of docetaxel resistance and promising molecular targets for PCa therapy.

## Materials and methods

### Cell lines, xenografts, and chemicals

Docetaxel-resistant (DOC) DU145 and PC3 PCa cell lines were generated as previously reported [17]. The docetaxel-sensitive PC346C and PC339 spheroid lines and their docetaxel-resistant derivatives, PC346C DOC and PC339 DOC were established from PCa patient-derived xenografts (PDXs) and propagated as described previously [18, 19]. All cell lines were maintained as defined in Supplementary Materials and Methods. Cells were routinely tested for mycoplasma contamination and authenticated using an AmpFLSTR Identifiler Plus PCR Amplification Kit (TFS, Czech Republic) to verify their origin.

### Antibody-based cell surface screening and spectral flow cytometry

The protocol published previously [20], also detailed in Supplementary Materials and Methods describes antibody-based cell surface screening and spectral flow cytometry procedures. Briefly, for high-throughput cell surface screening, cells were expanded, barcoded with CellTrace Violet and/or CellTrace DDAO (Far Red) amine-reactive fluorescent dyes, and subjected to the staining of 332 surface antigens using the LEGENDScreen Human Cell Screening PE Kit (cat. no. 700001; Biolegend, San Diego, CA, USA). Data were acquired on a FACSVerse (Becton Dickinson (BD), Franklin Lakes, NJ, USA), accessed with a Universal Loader. For multicolor spectral flow cytometry analysis, single-cell suspensions from PDXs and patient samples were subjected to red blood cells lysis and stained using a cocktail of fluorochrome-conjugated primary antibodies. Human cells within in vivo PDX models were selected based on anti-human CD298 positivity [21]. In patient samples, anti-human CD45, CD31, CD90 antibodies were used for the exclusion of leukocytes, endothelial-like cells, and stromal cells [22, 23]. Samples were analyzed using a SONY SP6800 Spectral Cell Analyzer (SONY, Japan). Acquired FCS files were exported and analyzed using FlowJo software (v10.0.7; BD). In all (spectral) flow cytometric experiments, dead cells were excluded from the analysis based on their positivity to LIVE/DEAD Fixable Dead Cell Stains (various dyes; Invitrogen, TFS). Cell aggregates and debris were excluded from the analysis based on a dual-parameter dot plot displaying the pulse ratio (signal height/y-axis vs. signal area/x-axis). A representative gating strategy is shown in Supplementary Fig. S1A. Dilution, clonality, fluorochrome information, and catalog numbers of the antibodies used for spectral flow cytometric analyses are provided in the Supplementary Materials and Methods and Supplementary Table S4. All antibodies were titrated before use or used as the manufacturer recommended.

### Patient-derived xenograft and prostate cancer tissue processing

PDX spheroids were harvested, dissociated, and inoculated subcutaneously into the right flank of six-week-old male severe combined immuno-deficient (SCID) hairless outbred (SHO) (Crl:SHO-*Prkdc*^*scid*^*Hr*^*hr*^) mice obtained from Charles River Laboratories (Wilmington, MA, USA). Each cohort (animals inoculated with a particular PDX model) contained four mice, corresponding to a total of 16 mice. Mice were euthanized with CO_2_ three weeks after inoculation when tumors reached 1 cm^3^ in volume, as determined by caliper measurements. Anesthesia was not administered during the experiment. Tumors were surgically excised, enzymatically dissociated using digestion medium, and stained. All European Union Animal Welfare lines (EU Directive 2010/63/EU for animal experiments) were followed. A detailed description of in vivo xenografts and tumor dissociation is provided in the Supplementary Materials and Methods. The animal experiments were approved by the Ethical Committee of IBP CAS and REKOZ, Academy of Sciences of the Czech Republic (AVCR 65/2016), supervised by the local ethical committee, and performed by certified individuals (SD and KS). Fresh PCa tumor samples (50–150 mg), evaluated by licensed pathologists, were obtained from the University Hospital Olomouc from patients undergoing robotic prostatectomy. All human tissue samples were obtained with the approval of the University Hospital Olomouc Ethical Committee (Ref. no. 83/19) from donors who provided written informed consent. Tissue samples were minced, enzymatically digested, and stained as described in Supplementary Materials and Methods.

### Extracellular vesicle isolation

To isolate extracellular vesicles (EVs), cells were expanded, harvested with 0.05% trypsin/EDTA, washed twice in PBS, and plated onto new plates into a vesicle-free medium. Supernatants were collected 72 h later. EVs were isolated by differential ultracentrifugation coupled with the sucrose cushion flotation step. Cryo-electron microscopy (cryo-EM) was used for EVs visualization. The size and concentration of the EVs were assessed using a multi-angled dynamic light scattering technique. The presence of EVs was validated using SDS-PAGE and western blotting. All procedure steps are described in detail in a previously published protocol [24] and in the Supplementary Materials and Methods.

### Liquid chromatography-mass spectrometry (LC-MS)

EV suspensions were solubilized and lysed in a solubilization buffer. The lysates were processed by filter-aided sample preparation. The resulting peptides were extracted into LC-MS vials, concentrated, and subjected to LC-MS analysis. Data were acquired in a data-independent acquisition mode (DIA). For a detailed sample preparation and LC-MS analysis description, see Supplementary Material and Methods. Four biological replicates were analyzed, with two being excluded due to quality control issues.

### Data analysis

As for the *Antibody-based cell surface screening*, cell lines were deconvolved based on the signal of the fluorescent barcode as described previously [25]. Both the medians of fluorescence and the percentage of positivity for the PE channel were exported and analyzed. For *Spectral flow cytometry analyses*, spectral overlaps were calculated and compensated based on spectral unmixing algorithms using the SONY SP6800 Software (SONY, Japan). All data, including multidimensional data from single-cell analyses and visualizations, were analyzed using FlowJo (v10.0.7; BD). Multiparametric data were processed using multidimensional reduction algorithms, including tSNE, UMAP, TriMap, FlowSOM, PhenoGraph, and X-shift. Statistical analyses were performed using Prism (v5, GraphPad, La Jolla, CA, USA). Clinical data sets (accession numbers GSE193898, GSE54460, and TCGA-PRAD) were retrieved via https://portal.gdc.cancer.gov/projects/TCGA-PRAD, GEO (NCBI), Oncomine (TFS), and the Genomic Data Commons Data Portal (NCI). Kaplan–Meier plots were assessed via customized analysis in the DriverDBv3 cancer omics database [26], and the median was set as a cutoff. The Biocarta Pathway (Harmonizome) was used for the correlation analysis of the z-score of sets of proteins employed within a particular pathway [27]. Heat maps were generated in Prism (v9, GraphPad, La Jolla, CA, USA). As for Mass spectrometry analysis of EVs, reported protein intensities were further processed using the software container environment (https://github.com/OmicsWorkflows), using the DIA-NN_PGs_LFQ_general_0.8 workflow which is available upon request. The workflow included removal of decoy his and contaminant protein groups. Only proteins identified at least on one proteotypic peptide were retained. Ratios between respective conditions were computed using MaxLFQ intensities, in case either numerator or denominator are missing, they were replaced by minimal value and reported separately. The mass spectrometry proteomics data have been deposited to the ProteomeXchange Consortium via the PRIDE [28] partner repository with the dataset identifier PXD050073.

## Results

### Docetaxel-resistant cells exhibit a unique surface molecule profile

**Fig. 1:**
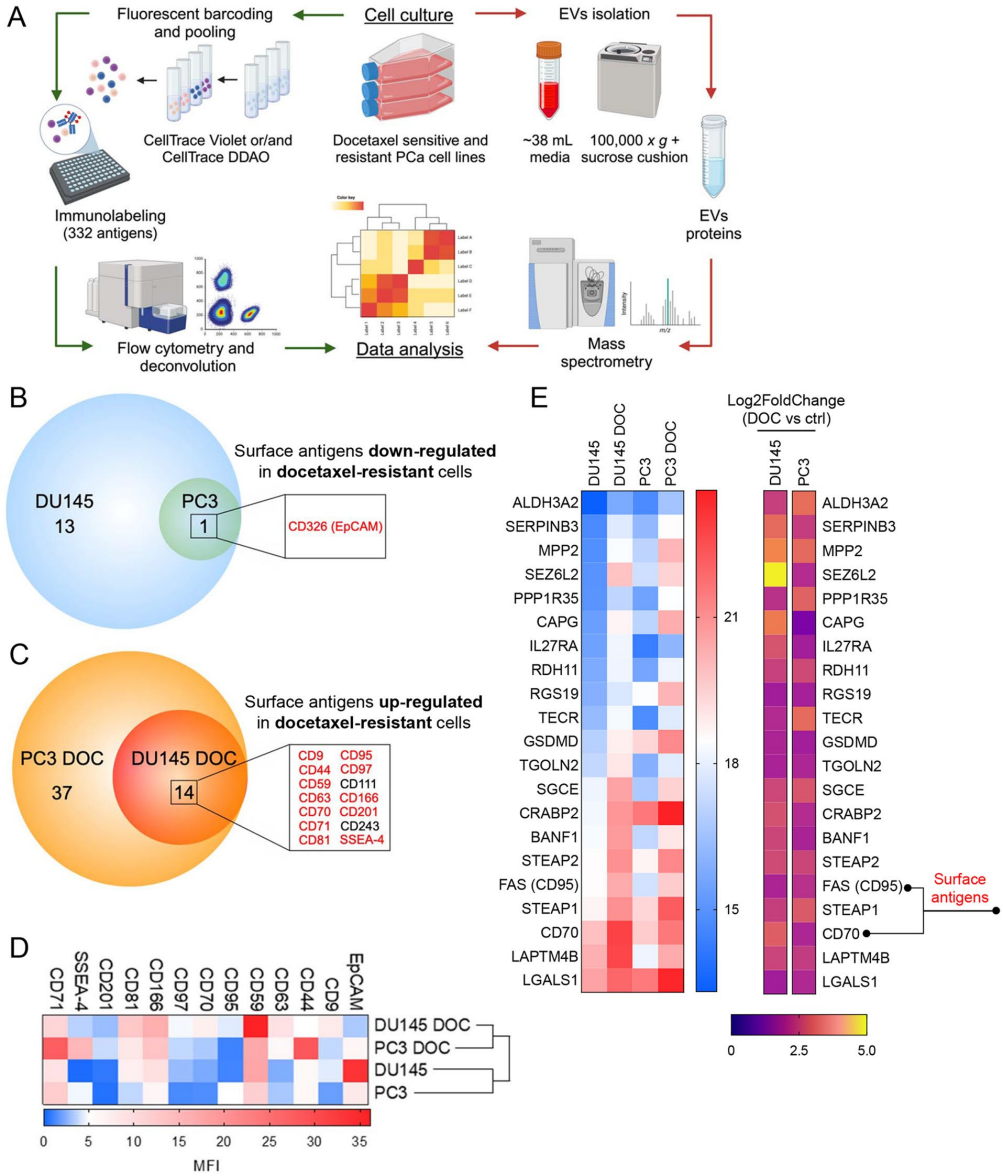
Profiling of surface antigens and EVs proteome in docetaxel-resistant PCa models in vitro. **A** The experimental workflow of high-throughput flow cytometry surface antigen screening and EVs isolation and mass spectrometry analysis of EVs proteome. Created with BioRender.com **B** Surface antigens downregulated on the surface of docetaxel-resistant cells. **C** Surface antigens upregulated on the surface of docetaxel-resistant cells. Surface antigens selected for further validation are highlighted in red. The threshold for selection was set as ≥ 1.5-fold change to docetaxel-sensitive counterparts. **D** Heatmap representing the expression of antigens selected based on the median fluorescence index (MFI). Heatmap portrays data from screening analysis (*n* = 1). **E** Heatmap displaying top 21 upregulated proteins within EVs from docetaxel-resistant cells analyzed by LC-MS (Log2 Fold Change ≥ 1.75) (*n* = 2)

To date, no experimental approach has been utilized to identify a specific surface fingerprint associated with docetaxel resistance. In this study, we adapted a previously introduced high-throughput flow cytometry screening platform for cell surfaceomes using a commercially available LEGENDScreen kit and fluorescent barcoding (Figs. 1A and S1A) [25]. Analysis of the surface molecule signature of two well-described docetaxel-resistant cell lines DU145 DOC and PC3 DOC revealed that out of 332 surface markers (Supplementary Tables S1 and S2), 81 antigens were expressed on the surface of at least one cell model (Supplementary Fig. S1B), 14 antigens were noticeably upregulated in both docetaxel-resistant cell lines, and only one antigen, EpCAM, was upregulated in both docetaxel-sensitive parental cell lines (Fig. 1B and C). From the list of surface antigens upregulated consistently in both docetaxel-resistant cell lines, the 12 most robustly upregulated antigens, CD9, CD44, CD59, CD63, CD70, CD71, CD81, CD95, CD97, CD166, CD201 and SSEA-4 (Fig. 1C) were, along with EpCAM, selected for further profiling (Supplementary Fig. S1C). Data analysis of the above-mentioned deregulated antigens showed docetaxel-resistant and docetaxel-sensitive cell lines clustering, highlighting similarities in the phenotypic features attributable to acquired docetaxel resistance (Fig. 1D).

### Proteomic analysis of extracellular vesicles indicates altered surface antigen expression of docetaxel-resistant cells

Extracellular vesicles (EVs) have recently been discovered to be critical modulators of resistance to cancer therapy [29]. Indeed, it has been summarized that EVs can function as sequesters of anticancer drugs, reducing their effectivity at target sites and thus serve as biomarkers for anticancer therapy response [30]. Here, we first isolated EVs from in vitro docetaxel-resistant and docetaxel-sensitive DU145 and PC3 cells and performed quality control to determine the presence, size, and mass of EVs. We employed cryo-EM (Fig. 1A and Supplementary Fig. S1A), dynamic light scattering (DLS) (Supplementary Fig. S2B), and western blot analysis of the proteins typically enriched in EVs (EV markers) markers CD9, FLOT1, and HSP70 as well as proteins reduced in EV isolates (EV negative markers) golgin-97 and Acetyl-α-Tubulin (Supplementary Fig. S2C) Next, we performed proteomic profiling of whole EVs and identified 21 proteins that were substantially enriched in both docetaxel-resistant models (Fig. 1E and Supplementary Fig. S2D). According to this analysis, two antigens, CD95 (Fas) and CD70, previously shown to be elevated on the surface of both docetaxel-resistant models, were also significantly upregulated in EVs isolated from both docetaxel-resistant models compared to docetaxel-sensitive models (Fig. 1E). In addition, EpCAM, CD9, CD44, CD63, and CD97 showed consistent trends of deregulation in both models (Supplementary Fig. S2E) with regard to the initial surface antigen screen (Fig. 1D). Other surface antigens displayed either no change or a trend in only one of the two docetaxel-resistant models (Supplementary Fig. S2D). These pilot data are intended to provide an initial, never published, overview of the composition of EVs originating from docetaxel-resistant prostate cancer cell lines and complement cellular surface profiling results. Overall, these data suggest that the surface antigen fingerprint unique to docetaxel-resistant cells is, to a large degree, reflected on the surface of EVs and thus has the potential to serve as a predictive parameter of docetaxel resistance.

### Validation of surface antigens in patient-derived xenografts in vivo confirms a unique surface profile for docetaxel-resistant cells

**Fig. 2:**
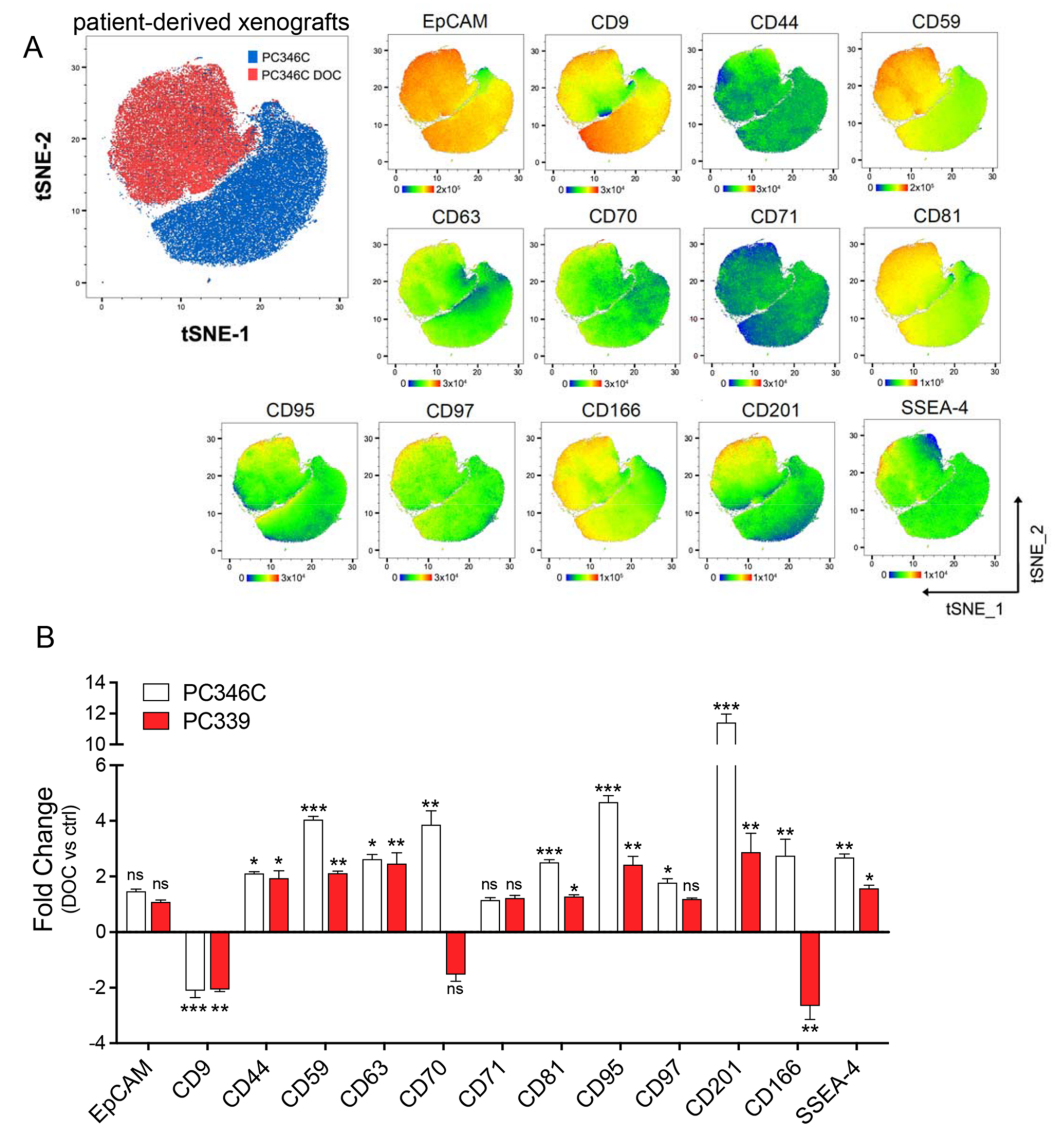
In vivo validation of docetaxel resistance-associated surface profile using PCa patient-derived xenografts. Tumors derived from PDXs were collected and dissociated, and single-cell suspensions were stained as described in Materials and Methods. **A** The main tSNE plot (left) shows the distribution of docetaxel-sensitive PC346C (blue) and docetaxel-resistant PC346C DOC (red) cells within the map. Related tSNE plots (right) illustrate the expression profile of particular antigens within the PC346C and PC346C DOC clusters. The color corresponds to the fluorescence intensity (red—high; blue—low). The range of fluorescence intensity related to a particular antigen is depicted below each plot. **B** Fold change (DOC vs. ctrl) expression of particular antigens in PDX models. The y-axis indicates the fold change of median fluorescence intensity. Data represent mean ± SEM from four independent tumors. ***, *P* < 0.0001; **, *P* < 0.01; *, *P* < 0.05 by unpaired *t*-test

Although our in vitro data portray a panel of surface antigens consistently deregulated in both docetaxel-resistant cell lines, these models lack lineage plasticity, a phenomenon that occurs within the primary tumor and contributes to intratumoral heterogeneity, EMT, diverse responses to therapy and taxane resistance [13, 31, 32]. Therefore, we validated surface markers identified by the in vitro screen in vivo, using preclinical docetaxel-resistant androgen-sensitive PDX PC346C and PC339 models [18, 19]. Positive selection of human cells from resected primary mouse tumors was achieved using human CD298 staining. Multicolor spectral flow cytometry assay followed by multidimensional data analysis using tSNE revealed partial heterogeneity with regard to selected surface antigens in both the PC346C and PC346C DOC PDX as well as PC339 and PC339 DOC models; nonetheless, docetaxel-resistant and docetaxel-sensitive cells clustered separately (Fig. 2A and Supplementary Fig. S3A). In addition to CD71 (level not detected) and EpCAM (level not changed), all antigens were expressed and were significantly altered in at least one of the two docetaxel-resistant PDXs as presented by fold change of median fluorescence intensity (Fig. 2B) as well as % positivity (Supplementary Fig. S3B). While the CD9 antigen was significantly reduced, CD44, CD59, CD63, CD81, CD95, CD97, CD201, and SSEA-4 antigens were commonly upregulated in both docetaxel-resistant PDXs as compared to their respective sensitive counterparts (Fig. 2A and B). CD70 and CD166, the two antigens that were linked to docetaxel resistance in vitro, were not considered for follow-up analysis due to inconsistent surface expression between both PDX pairs (Fig. 2B). Collectively, we established a 9-molecule surface fingerprint shared between the most relevant and available docetaxel-resistant models in vitro and in vivo.

### Docetaxel resistance-related fingerprint pre-exists in primary prostate cancer cells

**Fig. 3:**
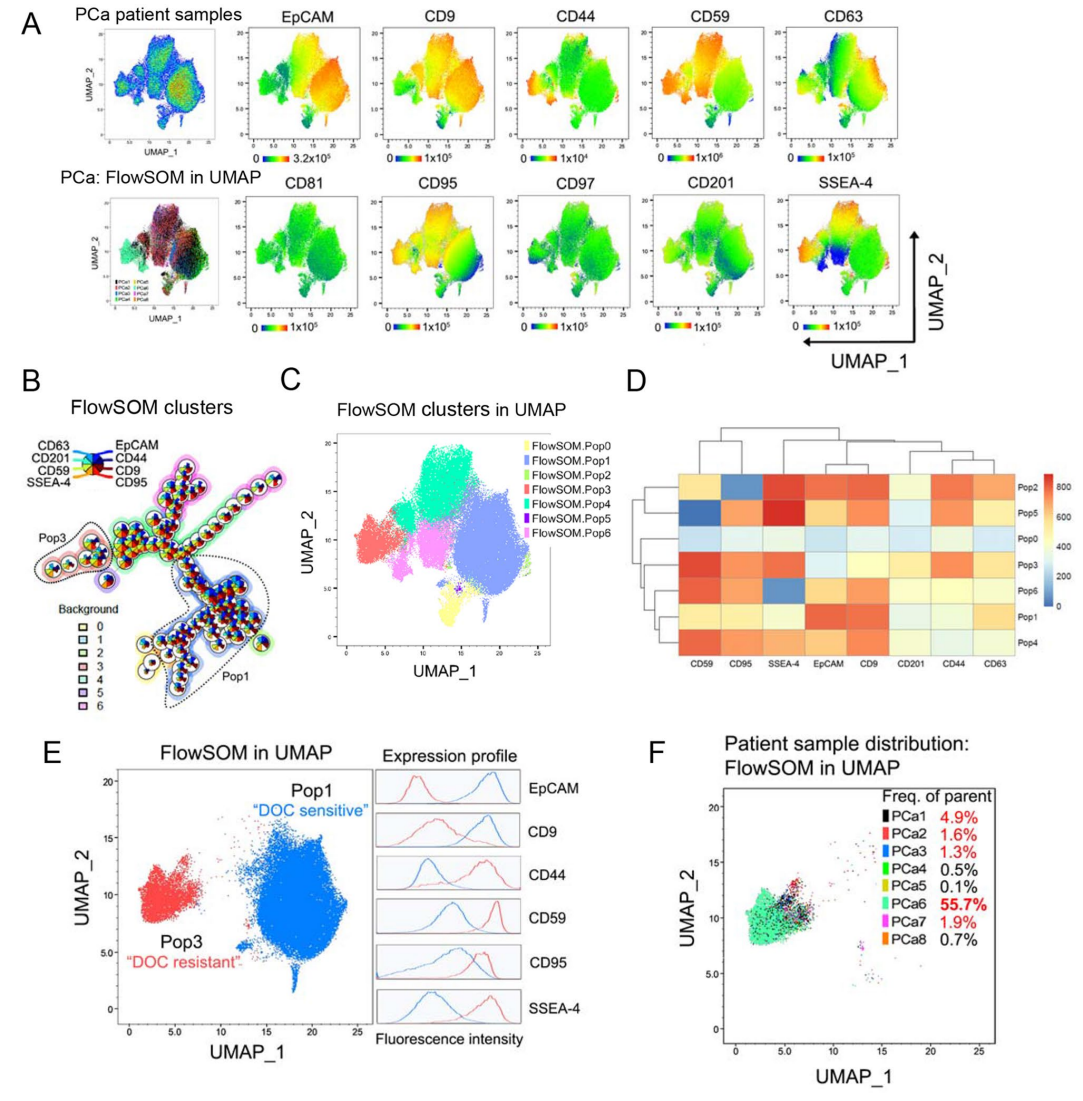
Inspection of the expression of selected antigens in dissociated primary PCa samples. Fresh PCa samples (*n* = 8) collected from eight patients undergoing surgical prostatectomy were minced, enzymatically digested, and stained as described in Supplementary Materials and Methods. **A** The UMAP plot in pseudocolor (top, left) indicates the distribution of cells from eight PCa patient samples within the map based on the expression of selected antigens. The FlowSOM in UMAP visualization (left, down) indicates the distribution of patient samples within the map determined by FlowSOM. Each color denotes a different patient sample. Related UMAP plots (right) illustrate the expression profile of particular surface antigens within the clusters. The color corresponds to the fluorescence intensity (red—high; blue—low). The range of fluorescence intensity related to a particular antigen is depicted below each plot. **B** FlowSOM algorithm illustrating clustering of cell subpopulation based on the eight markers with detectable expression in patient samples. FlowSOM is visualized as nodes, where each node represents a cluster of a specific subpopulation of cells, and pie charts of the node reflect the contribution of different markers onto the phenotype of the cell cluster. **C** UMAP visualization of FlowSOM clustering. **D** Heatmap corresponding to the FlowSOM clustering, depicting the expression of surface antigens within each subpopulation. **E** UMAP visualization of selected Pop3 “DOC resistant” reflecting the fingerprint of docetaxel-resistant PDXs and Pop1 “DOC sensitive” reflecting the fingerprint of docetaxel-sensitive PDXs, determined by FlowSOM. Related expression profiles of the six most deregulated antigens are displayed by histograms on the right (red, “docetaxel-resistant”; blue, “docetaxel-sensitive”). **F** UMAP visualization of the distribution of patient samples within the “resistant” population of cells determined by FlowSOM. Each color denotes a different patient sample. The values in the legend refer to the % of cells belonging to the “resistant” population from the overall number of cells in each patient sample

In the follow-up investigation, we profiled a cohort of eight untreated primary PCa patient samples using the previously determined 9-molecule surface fingerprint of docetaxel-resistant cells combined with the epithelial marker EpCAM (Supplementary Table S3). The specimens were collected by radical prostatectomy, dissociated using a well-established protocol, and stained as described in the Supplementary Material and Methods. We used human CD45, CD31, and CD90 staining to discriminate between leukocytes, endothelial cells, and cancer-associated stromal cells, respectively, and investigated the expression of selected antigens in primary tumor epithelial cells only. Advanced, multidimensional data analysis of patient samples revealed heterogeneity in the expression of all antigens, except for CD81 and CD97, which were not expressed in any subpopulation of the patient samples and thus further considered as not clinically relevant (Fig. 3A). Unsupervised FlowSOM clustering algorithms uncovered a cell subpopulation (Pop3) that was characterized by high expression of CD44, CD59, CD95, and SSEA-4 and low levels of EpCAM and CD9, reflecting the surface signature of previously examined docetaxel-resistant PDX models, PC346C DOC and PC339 DOC (Fig. 3B–D and Supplementary Fig. S4A). In addition, our data revealed a cell subpopulation (Pop1) with the opposite phenotype, expressing low CD44, CD59, CD95, and SSEA-4, and high EpCAM and CD9 levels, resembling the surface antigen expression profiles shared across all previously inspected docetaxel-sensitive cell lines and PDX models (Fig. 3B–D and Supplementary Fig. S4A). Due to very low to absent expression of CD63 and CD201 within these two patient sample subpopulations (Fig. 3A and D), we disregarded these antigens from further investigations. In light of previous results, a 6-molecule surface fingerprint containing EpCAM, CD9, CD44, CD59, CD95, and SSEA-4 was examined in the Pop1 and Pop3 PCa patient sample subpopulations to prove its potential as a comprehensive fingerprint for prediction of docetaxel resistance or sensitivity in PCa patients (Fig. 3E and Supplementary Fig. S4B). The presence of Pop3 subpopulation, based on the 6-molecule surface fingerprint, was further validated to exist using multiple clustering and projection algorithms enabling multidimensional data reduction (Supplementary Fig. S5). Importantly, we could show that the Pop3 subpopulation, reflecting the docetaxel-resistant phenotype, was present in 5 out of 8 patient samples as overt or rare subpopulation ranging from 1.3% (PCa3) to 55.7% (PCa6), illustrating patient-to-patient variability (Fig. 3F). In conclusion, based on preclinical models of docetaxel resistance and primary docetaxel-naïve PCa patient samples, we propose a 6-molecule surface fingerprint composed of EpCAM, CD9, CD44, CD59, CD95, and SSEA-4 as a candidate set of surface antigens that may potentially predict the response to docetaxel in PCa patients before therapy.

### CD95 and SSEA-4 are potential biomarkers for docetaxel resistance and poor survival prognosis in prostate cancer patients

**Fig. 4:**
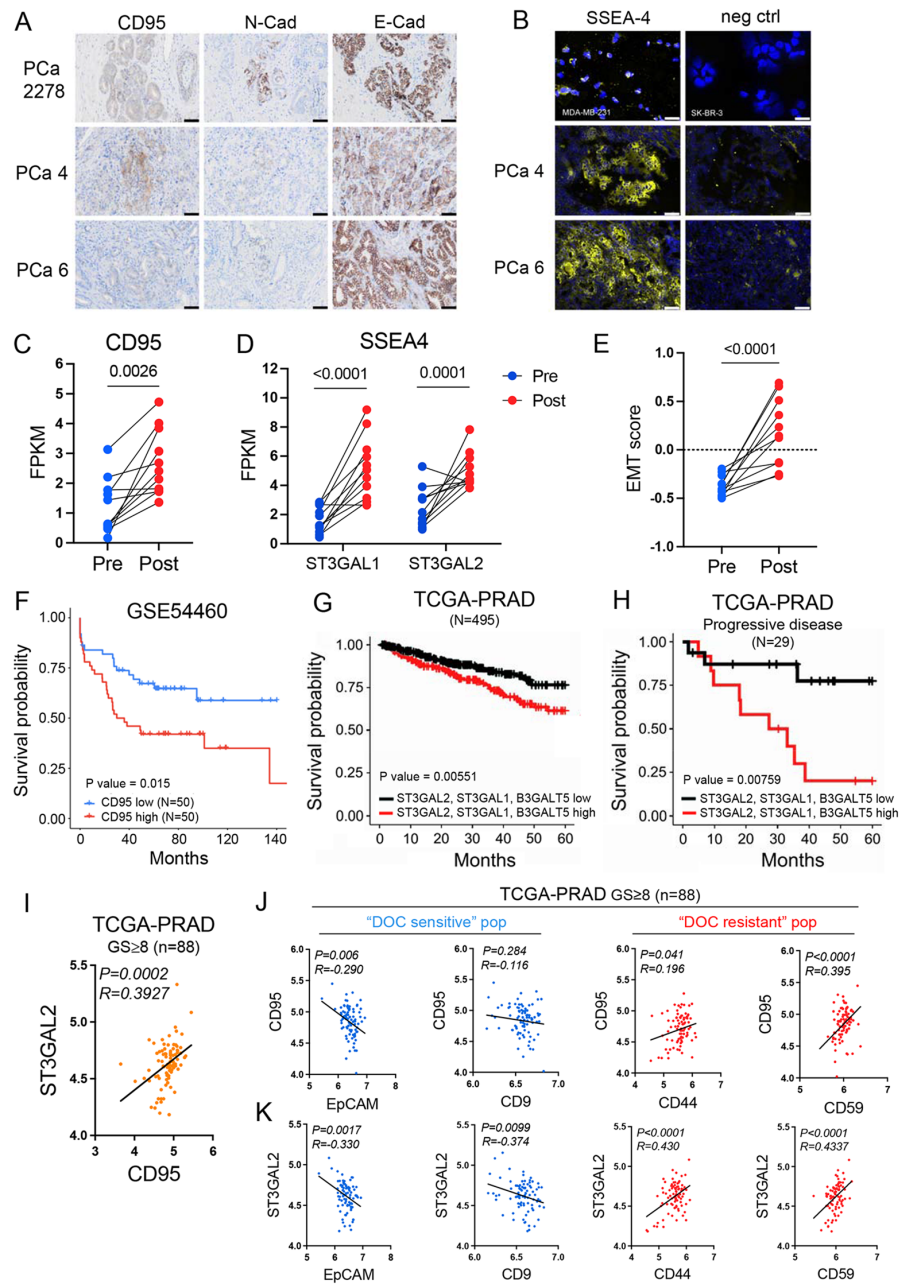
Assessment of CD95 and SSEA-4 expression in the context of docetaxel resistance within clinical sample setting. **A** Immunohistochemistry staining of CD95, N-Cadherin, and E-cadherin on selected patient samples. Sample “PCa 2278” has been used as positive control for expression of all 3 antigens. **B** Immunofluorescent staining of SSEA-4 in selected patient samples. Validation of the SSEA-4 staining procedure is provided with MDA-MB-231 positive and SK-BR-3 negative control; SSEA4 expression on these cells was analyzed by flow cytometry, data not shown). (C, D) Expression of **C** CD95 and **D** SSEA-4 synthesis-related enzymes ST3GAL1 and ST3GAL2 in patient samples pre- or post-docetaxel-based therapy (*n* = 11). **E** EMT score on the scale from − 1 (Epi) to 1 (Mes) computed for pre- and post-docetaxel-based therapy patient samples using a two-sample Kolmogorov–Smirnov test [39] (*n* = 11). **F** Survival probability in a cohort of PCa patients according to high or low CD95 expression (GSE54460, *n* = 50), **G, H** Survival probability according to high or low ST3GAL2, ST3GAL1 and B3GALT5 expression levels (TCGA) in a cohort of all (n = 495) or progressive disease only (n = 29) PCa patients, respectively. **I** Correlation analysis of ST3GAL2 and CD95 expression in patients with high-grade (GS ≥ 8) PCa disease (n = 88) (TCGA). **J, K** Correlation analysis of CD95, ST3GAL2 and surface antigens from the PDX fingerprints in the “DOC sensitive” (Pop1; in blue) or “DOC resistant” subpopulation (Pop3; in red), respectively (see text for further details)

Considering previous data, CD95 and SSEA-4 were some of the most differentially expressed surface antigens compared to docetaxel-resistant and docetaxel-sensitive cell lines and PDXs and heterogeneously expressed in primary PCa patient samples. Therefore, we aimed to examine whether their elevated levels alone would reflect docetaxel resistance and poor disease outcomes in patients with PCa. Here, we provide an illustrative set of primary PCa samples that demonstrates substantial heterogeneity in CD95 and SSEA4 expression (Fig. 4A and B and Supplementary Fig. S6). This phenomena is accompanied by heterogeneous N-cadherin and consistent E-cadherin expression, which is commonly used for EMT assessment in PCa [33], within similar areas of tissue from two PCa patient samples previously used for surface profiling (Fig. 4A and B). Furthermore, we retrieved data from a publicly available RNA-seq database, which included 11 paired pre- and post-docetaxel-based PCa tumor samples. Our data analysis showed a significant increase in CD95 expression post-therapy in all 11 patients (Fig. 4C). In addition, the expression of two fundamental enzymes involved in glycosphingolipid SSEA-4 synthesis, ST3GAL2 and ST3GAL1 (Supplementary Fig. S7A), was significantly upregulated in all post-therapy samples compared to that in pre-therapy samples (Fig. 4D). Notably, the other antigens from the 6-molecule fingerprint, EpCAM, CD9, CD44, and CD59, showed a similar trend in expression change as that previously observed in preclinical models of docetaxel resistance and primary PCa patient samples (Supplementary Fig. S7B). These results together corroborate the clinical relevance of these surface antigens in docetaxel-resistant PCa.

CD95/CD95L pathway modulation and activation has been associated with the increased killing of PCa cells and enhanced sensitivity to chemotherapy-induced apoptosis [34]. Therefore, we tested whether the upregulation of CD95 in docetaxel-resistant PCa cells is associated with increased sensitivity to CD95L. We analyzed cell viability using the CyQuant assay in docetaxel-resistant and -sensitive PC3 cells treated with increasing concentrations of CD95L and showed that docetaxel-resistant PC3 cells exhibit significantly higher sensitivity to CD95L than their sensitive counterparts (Supplementary Fig. S7C).

Given our data, previously published studies on PCa cells indicating EMT transition as a function of docetaxel resistance [17, 35, 36] and the link between EMT and increased expression of CD95 and SSEA-4 [37, 38], we computed the EMT score reflecting the extent to which cells display epithelial or mesenchymal phenotype on a scale from −1 (Epi) to 1 (Mes), in all pre- and post-docetaxel-based therapy samples using a two-sample Kolmogorov–Smirnov test [39]. This revealed an EMT switch and gain of mesenchymal features post-docetaxel-based therapy in all 11 matched patient samples (Fig. 4E). Furthermore, data from publicly available datasets showed that high expression of CD95 (Fig. 4F), as well as ST3GAL2, ST3GAL1, and B3GALT5 (essential for synthesis of SSEA-4 precursor, SSEA-3) (Fig. 4G and Supplementary Fig. S7A), correlated with an unfavorable prognosis determined by survival probability, particularly in a cohort of pre-docetaxel patients with progressive disease (Fig. 4H). Moreover, ST3GAL2 expression was also positively correlated with the z-score of a gene set associated with resistance to anti-microtubule agents, notably in a cohort of docetaxel-naïve patients with high-grade disease (GS ≥ 8) (Supplementary Fig. S7D and S7E). Likewise, analysis of TCGA data of primary untreated patients demonstrated a positive correlation between ST3GAL2 and CD95 (Fig. 4I and Supplementary Fig. S7F) and a negative correlation between CD95 and ST3GAL2 with EpCAM and CD9 expression along with a positive correlation with CD44 and CD59 expression (Fig. 4J and K and Supplementary Fig. S7G and S7H). Notably, in general, stronger correlations were observed in patients with high-grade disease (GS ≥ 8), emphasizing the importance of these markers in advanced PCa (Fig. 4I–K).

These analyses endorse further clinical relevance of the 6-molecule surface fingerprint as a potential predictive tool for docetaxel resistance in patients with PCa.

## Discussion

Despite initial therapy success, PCa progresses to a life-threatening advanced stage of metastatic disease which remains the second major cause of cancer death in men [40, 41]. Docetaxel represents the backbone treatment against advanced PCa [42–44]. Moreover, recent therapeutic approaches have utilized early-stage administration of docetaxel alone as well as in combination with hormonal therapy or androgen-deprivation in hormone-sensitive PCa patients [45, 46]. However, the clinical utility of docetaxel is substantially compromised in a significant proportion of men due to the development of acquired therapy resistance [47]. Although docetaxel-resistance represents a critical hurdle in PCa therapy, changes in the transcriptome and proteome of docetaxel-resistant cells that would help to recognize these resistance pathways already before therapy and which may constitute potential targets for treatment remain largely unknown. Here, we examined cancer cell phenotype linked to docetaxel resistance using single-cell analysis of the surfaceome signature in vitro and in vivo and follow-up validation using PCa patient samples and publicly available clinical datasets.

To identify a unique surface signature of docetaxel resistance, we used well-described docetaxel-resistant in vitro models DU145 DOC and PC3 DOC [17]. Previous studies using these cell lines have suggested that EMT, including upregulation of vimentin, CD44, or ZEB1 and loss of E-cadherin, is a potential driving mechanism of docetaxel resistance [17, 35, 36]. Additionally, CD44 was also established as a driver of invasion and migration and its overexpression was associated with therapy resistance and neuroendocrine-like phenotype in PCa [48, 49]. In concordance with these reports, our screening revealed downregulation of the epithelial marker EpCAM and an upregulation of the mesenchymal marker CD44 in docetaxel-resistant models, suggesting an EMT switch. In addition, 11 antigens previously never directly linked to docetaxel resistance *per se* were consistently upregulated in DU145 DOC and PC3 DOC cells.

Emerging evidence shows that resistance to paclitaxel or docetaxel, both anti-microtubule drugs, can be transferred to neighboring cancer cells within one tumor, or potentially also to metastatic cells at distant sites through cancer cell-shedded EVs (also known as exosomes) that carry cellular information crucial to reprogram the pre-metastatic niche as well as therapy-sensitive cancer cells and profile therapy resistance [50–54]. EVs can act as active transporters of docetaxel out of cancer cells, thus decreasing intracellular retention and impairing its effectivity [55]. Our analysis of EVs in both DU145 and PC3 cells showed that proteome profiles of docetaxel-resistant and docetaxel-sensitive cells differ significantly. Focusing specifically on the surface fingerprints, the EMT traits of docetaxel-resistant cells are reflected on the surface of EVs (Supplementary Fig. S2E). Such features may be essential for regulating the distant metastatic cell phenotype, growth, and sensitivity to therapy [50, 56]. In addition, we show that several other antigens presented on the surface of docetaxel-resistant cells are also captured in EVs shed by these cells (Supplementary Figure S2E). Although further experiments are necessary to prove the functional role of EVs in docetaxel resistance, these data support the potential use of EVs as a diagnostic tool for docetaxel-resistant PCa in blood.

To further validate the relevance of the identified surface antigens altered in docetaxel resistance in vivo, we utilized docetaxel-resistant PC346C DOC and PC339 DOC PDXs [18, 19]. We could confirm a similar up- or down-regulated switch in the 9 surface antigens in these PDX as identified in PC3 and DU145 cell lines. Follow-up validation of the pre-clinically defined surface fingerprint in a cohort of PCa patient samples resulted in a 6-molecule surface fingerprint composed of EpCAM, CD9, CD44, CD59, CD95, and SSEA-4. This fingerprint served as a template for the identification of Pop1 and Pop3 subpopulations, which, based on the surface antigen profile, refers to docetaxel resistance or sensitivity in vivo, respectively, and hence may characterize docetaxel-resistant or docetaxel-sensitive cancer cell subpopulations in PCa tumors.

While direct evidence of molecular interaction between antigens of the 6-molecule surface fingerprint is unknown, there are indications that some molecules may participate in related pathways and processes, particularly in the context of therapy resistance. For instance, SSEA-4 that was upregulated in all in vitro and in vivo models of docetaxel resistance and has been previously associated with tumorigenicity, chemoresistance, and mesenchymal features [57–59], was found to be co-expressed with mesenchymal marker CD44 in a subpopulation of tumor cells responsible for cancer stem-like cell characteristics, malignant behavior, and worse overall survival [60–62]. Also, SSEA-4-positive cancer stem cells exhibited activation of PI3K/Akt pathway [37] which has been previously shown to enhance the transcriptional activity of nuclear factor-kappa B (NF-κB) [63], a key regulator of tumor necrosis factor receptor CD95, a complement regulatory protein CD59 expression as well as multidrug resistance [63–65]. In a feedback loop, CD95 was portrayed to phosphorylate PI3K/Akt and promote pro-survival signaling [66]. Therefore, hypothetically, in a scenario where SSEA-4-positive PCa cancer cells are resistant to docetaxel treatment, CD95 and CD59 will be dysregulated as well. While CD95 will protect PCa cells against apoptosis, CD59, in turn, may contribute to immune evasion by protecting SSEA-4-positive PCa cells from complement-mediated lysis, thus promoting their therapy resistance and survival. Such a scenario would explain increased SSEA-4, CD95 as well as CD59 in all docetaxel-resistant PCa models used in this study. It would also align with studies demonstrating strong increase of CD95 expression in primary PCa cells after docetaxel treatment [34], positive correlation of higher levels of soluble CD95, docetaxel resistance and shorter cancer-specific survival in PCa patients [67], as well as incline in CD59 expression in treatment naïve PCa correlating with early biochemical relapse and worse prognosis [68]. In addition, given the increased sensitivity of docetaxel-resistant PC3 cells to CD95L we assume that treatment with this ligand may rewire the non-canonical, pro-survival CD95 signaling in docetaxel-resistant cells and induce canonical, apoptosis-triggering CD95L-CD95 signaling, representing potential therapeutic avenue for the therapy of advanced PCa.

It is important to note that the above-mentioned scenario is rather speculative, and direct evidence of a molecular interaction is lacking. Further research is therefore needed to elucidate potential crosstalk and functional relationships between these antigens in docetaxel resistance and cancer biology in general. The 6-molecule surface fingerprint expression profile also displayed high patient-to-patient variability, which may have been caused by different patient’s disease stages and clinical history variations. Notably, the distribution and abundance of the cell subpopulation with docetaxel-resistant signatures were not dependent on the disease stage or anticancer therapy (Supplementary Table S3). This observation may suggest that pre-existing intratumoral heterogeneity shapes docetaxel resistance due to early tumor plasticity rather than *de novo* arising from drug-tolerant “persister” cells.

In summary, we propose a 6-molecule surface fingerprint with the potential to identify docetaxel-resistant cells in patients early. The surface fingerprint’s more detailed classification of these cell subpopulations may reveal the mechanism(s) of docetaxel resistance, which may guide new approaches to targeting docetaxel resistance and improve advanced PCa therapy.

## Electronic supplementary material

Below is the link to the electronic supplementary material.

Supplementary Material 1

Supplementary Material 2

Supplementary Material 3

## Data Availability

The mass spectrometry proteomics data have been deposited to the ProteomeXchange Consortium via the PRIDE partner repository with the dataset identifier PXD050073.

## Abbreviations

CAR: Chimeric antigen receptor
DLS: Dynamic light scattering
DOC: Docetaxel-resistant
EMT: Epithelial-to-mesenchymal transition
PCa: Prostate cancer
PDXs: Patient-derived xenografts
PI3K: Phosphoinositide 3-kinase
SSEA-4: Stage-specific embryonic antigen-4
ST3GAL: ST3 beta-galactoside alpha-2,3-sialyltransferase

## References

[1] K. Komura, S.H. Jeong, K. Hinohara, F. Qu, X. Wang, M. Hiraki, H. Azuma, G.S. Lee, P.W. Kantoff, C.J. Sweeney, Proc. Natl. Acad. Sci. U. S. A. **113**, 6259–6264 (2016). 10.1073/pnas.1600420113 10.1073/pnas.1600420113 PMC4896695 27185910

[2] I. Dagogo-Jack, A.T. Shaw, Nat. Rev. Clin. Oncol. **15**, 81–94 (2018). 10.1038/nrclinonc.2017.166 10.1038/nrclinonc.2017.166 29115304

[3] M. Gerlinger, A.J. Rowan, S. Horswell, M. Math, J. Larkin, D. Endesfelder, E. Gronroos, P. Martinez, N. Matthews, A. Stewart, P. Tarpey, I. Varela, B. Phillimore, S. Begum, N.Q. McDonald, A. Butler, D. Jones, K. Raine, C. Latimer, C.R. Santos, M. Nohadani, A.C. Eklund, B. Spencer-Dene, G. Clark, L. Pickering, G. Stamp, M. Gore, Z. Szallasi, J. Downward, P.A. Futreal, C. Swanton, N. Engl. J. Med. **366**, 883–892 (2012). 10.1056/NEJMoa1113205 10.1056/NEJMoa1113205 PMC4878653 22397650

[4] A.B. Turke, K. Zejnullahu, Y.L. Wu, Y. Song, D. Dias-Santagata, E. Lifshits, L. Toschi, A. Rogers, T. Mok, L. Sequist, N.I. Lindeman, C. Murphy, S. Akhavanfard, B.Y. Yeap, Y. Xiao, M. Capelletti, A.J. Iafrate, C. Lee, J.G. Christensen, J.A. Engelman, P.A. Janne, Cancer Cell **17**, 77–88 (2010). 10.1016/j.ccr.2009.11.022 10.1016/j.ccr.2009.11.022 PMC2980857 20129249

[5] H.E. Bhang, D.A. Ruddy, V. Krishnamurthy Radhakrishna, J.X. Caushi, R. Zhao, M.M. Hims, A.P. Singh, I. Kao, D. Rakiec, P. Shaw, M. Balak, A. Raza, E. Ackley, N. Keen, M.R. Schlabach, M. Palmer, R.J. Leary, D.Y. Chiang, W.R. Sellers, F. Michor, V.G. Cooke, J.M. Korn, F. Stegmeier, Nat. Med. **21**, 440–448 (2015). 10.1038/nm.3841 10.1038/nm.3841 25849130

[6] B. Wu, X. Lu, H. Shen, X. Yuan, X. Wang, N. Yin, L. Sun, P. Shen, C. Hu, H. Jiang, D. Wang, Int. J. Cancer **146**, 3369–3378 (2020). 10.1002/ijc.32961 10.1002/ijc.32961 32159858

[7] S.S. Yadav, J.A. Stockert, V. Hackert, K.K. Yadav, A.K. Tewari, Urol. Oncol. **36**, 349–360 10.1016/j.urolonc.2018.05.008 10.1016/j.urolonc.2018.05.008 29887240

[8] Y.C.S. Ramon, M. Sese, C. Capdevila, T. Aasen, L. De Mattos-Arruda, S.J. Diaz-Cano, J. Hernandez-Losa, J. Castellvi, J. Mol. Med. (Berl) **98**, 161–177 (2020). 10.1007/s00109-020-01874-2 10.1007/s00109-020-01874-2 PMC7007907 31970428

[9] A. Quintanal-Villalonga, J.M. Chan, H.A. Yu, D. Pe’er, C.L. Sawyers, T. Sen, C.M. Rudin, Nat. Rev. Clin. Oncol. **17**, 360–371 (2020). 10.1038/s41571-020-0340-z 10.1038/s41571-020-0340-z PMC7397755 32152485

[10] T. Graf, T. Enver, Nature **462**, 587–594 (2009). 10.1038/nature08533 10.1038/nature08533 19956253

[11] S. Laudato, A. Aparicio, F.G. Giancotti, Trends Cancer **5**, 440–455 (2019). 10.1016/j.trecan.2019.05.008 10.1016/j.trecan.2019.05.008 PMC6658113 31311658

[12] C.E. Meacham, S.J. Morrison, Nature **501**, 328–337 (2013). 10.1038/nature12624 10.1038/nature12624 PMC4521623 24048065

[13] H. Beltran, A. Hruszkewycz, H.I. Scher, J. Hildesheim, J. Isaacs, E.Y. Yu, K. Kelly, D. Lin, A. Dicker, J. Arnold, T. Hecht, M. Wicha, R. Sears, D. Rowley, R. White, J.L. Gulley, J. Lee, M. Diaz Meco, E.J. Small, M. Shen, K. Knudsen, D.W. Goodrich, T. Lotan, A. Zoubeidi, C.L. Sawyers, C.M. Rudin, M. Loda, T. Thompson, M.A. Rubin, A. Tawab-Amiri, W. Dahut, P.S. Nelson, Clin. Cancer. Res. **25**, 6916–6924 (2019). 10.1158/1078-0432.CCR-19-1423 10.1158/1078-0432.CCR-19-1423 PMC6891154 31363002

[14] P.M. Schnepp, G. Shelley, J. Dai, N. Wakim, H. Jiang, A. Mizokami, E.T. Keller, Mol. Cancer Res. **18**, 1290–1301 (2020). 10.1158/1541-7786.MCR-20-0051 10.1158/1541-7786.MCR-20-0051 PMC7483674 32513898

[15] D. Keresztes, A. Csizmarik, N. Nagy, O. Modos, T. Fazekas, T. Bracht, B. Sitek, K. Witzke, M. Puhr, S. Sevcenco, G. Kramer, S. Shariat, Z. Kuronya, L. Takacs, I. Tornyi, J. Lazar, B. Hadaschik, A. Laszik, M. Szucs, P. Nyirady, T. Szarvas, J. Cell Mol. Med. **26**, 1332–1337 (2022). 10.1111/jcmm.17141 10.1111/jcmm.17141 PMC8831956 34970839

[16] J. Zhang, S. Li, J. Zhang, W. Zhang, J. Jiang, H. Wu, E. Wu, Y. Feng, L. Yang, Z. Li, Cancer Lett. **545**, 215829 (2022). 10.1016/j.canlet.2022.215829 10.1016/j.canlet.2022.215829 35868534

[17] M. Puhr, J. Hoefer, G. Schafer, H.H. Erb, S.J. Oh, H. Klocker, I. Heidegger, H. Neuwirt, Z. Culig, Am. J. Pathol. **181**, 2188–2201 (2012). 10.1016/j.ajpath.2012.08.011 10.1016/j.ajpath.2012.08.011 23041061

[18] E.S. de Morree, R. Bottcher, R.J. van Soest, A. Aghai, C.M. de Ridder, A.A. Gibson, R.H. Mathijssen, H. Burger, E.A. Wiemer, A. Sparreboom, R. de Wit, W.M. van Weerden, Br. J. Cancer **115**, 674–681 (2016). 10.1038/bjc.2016.251 10.1038/bjc.2016.251 PMC5023781 27537383

[19] W.M. van Weerden, C. Bangma, R. de Wit, Br. J. Cancer **100**, 13–18 (2009). 10.1038/sj.bjc.6604822 10.1038/sj.bjc.6604822 PMC2634688 19088719

[20] S. Drapela, R. Fedr, O. Vacek, J. Remsik, K. Soucek, Methods Mol. Biol. **2543**, 99–111 (2022). 10.1007/978-1-0716-2553-8_9 10.1007/978-1-0716-2553-8_9 36087262

[21] F.J. Hartmann, E.F. Simonds, S.C. Bendall, Sci. Rep. **8**, 10770 (2018). 10.1038/s41598-018-28791-2 10.1038/s41598-018-28791-2 PMC6050312 30018331

[22] M. Bartoschek, N. Oskolkov, M. Bocci, J. Lovrot, C. Larsson, M. Sommarin, C.D. Madsen, D. Lindgren, G. Pekar, G. Karlsson, M. Ringner, J. Bergh, A. Bjorklund, K. Pietras, Nat. Commun. **9**, 5150 (2018). 10.1038/s41467-018-07582-3 10.1038/s41467-018-07582-3 PMC6279758 30514914

[23] D. Boral, M. Vishnoi, H.N. Liu, W. Yin, M.L. Sprouse, A. Scamardo, D.S. Hong, T.Z. Tan, J.P. Thiery, J.C. Chang, D. Marchetti, Nat. Commun. **8**, 196 (2017). 10.1038/s41467-017-00196-1 10.1038/s41467-017-00196-1 PMC5543046 28775303

[24] V. Pospichalova, J. Svoboda, Z. Dave, A. Kotrbova, K. Kaiser, D. Klemova, L. Ilkovics, A. Hampl, I. Crha, E. Jandakova, L. Minar, V. Weinberger, V. Bryja, J. Extracell. Vesicles **4**, 25530 (2015). 10.3402/jev.v4.25530 10.3402/jev.v4.25530 PMC4382613 25833224

[25] J. Remsik, R. Fedr, J. Navratil, L. Bino, E. Slabakova, P. Fabian, M. Svoboda, K. Soucek, Br. J. Cancer **118**, 813–819 (2018). 10.1038/bjc.2017.497 10.1038/bjc.2017.497 PMC5886127 29462126

[26] S.H. Liu, P.C. Shen, C.Y. Chen, A.N. Hsu, Y.C. Cho, Y.L. Lai, F.H. Chen, C.Y. Li, S.C. Wang, M. Chen, I.F. Chung, W.C. Cheng, Nucleic Acids Res. **48**, D863–D870 (2020). 10.1093/nar/gkz964 10.1093/nar/gkz964 PMC7145679 31701128

[27] A.D. Rouillard, G.W. Gundersen, N.F. Fernandez, Z. Wang, C.D. Monteiro, M.G. McDermott, A. Ma’ayan, Database (Oxford) **2016**, (2016). 10.1093/database/baw100 10.1093/database/baw100 PMC4930834 27374120

[28] Y. Perez-Riverol, J. Bai, C. Bandla, D. Garcia-Seisdedos, S. Hewapathirana, S. Kamatchinathan, D.J. Kundu, A. Prakash, A. Frericks-Zipper, M. Eisenacher, M. Walzer, S. Wang, A. Brazma, J.A. Vizcaino, Nucleic Acids Res. **50**, D543–D552 (2022). 10.1093/nar/gkab1038 10.1093/nar/gkab1038 PMC8728295 34723319

[29] F. Fontana, E. Carollo, G.E. Melling, D.R.F. Carter, Cancers (Basel) **13**, (2021). 10.3390/cancers13040749 10.3390/cancers13040749 PMC7916933 33670185

[30] S. Maacha, A.A. Bhat, L. Jimenez, A. Raza, M. Haris, S. Uddin, J.C. Grivel, Mol. Cancer **18**, 55 (2019). 10.1186/s12943-019-0965-7 10.1186/s12943-019-0965-7 PMC6441157 30925923

[31] R. Tiwari, N. Manzar, B. Ateeq, Front. Mol. Biosci. **7**, 130 (2020). 10.3389/fmolb.2020.00130 10.3389/fmolb.2020.00130 PMC7365877 32754615

[32] N. Jimenez, O. Reig, R. Montalbo, M. Mila-Guasch, L. Nadal-Dieste, G. Castellano, J.J. Lozano, I. Victoria, A. Font, A. Rodriguez-Vida, J. Carles, C. Suarez, M. Domenech, N. Sala-Gonzalez, P.L. Fernandez, L. Rodriguez-Carunchio, S. Diaz, A. Prat, M. Marin-Aguilera, B. Mellado, Front. Oncol. **10**, 594023 (2020). 10.3389/fonc.2020.594023 10.3389/fonc.2020.594023 PMC7667288 33224888

[33] K. Kolijn, E.I. Verhoef, G.J. van Leenders, Oncotarget **6**, 24488–24498 (2015). 10.18632/oncotarget.4177 10.18632/oncotarget.4177 PMC4695200 26041890

[34] J.C. Symes, M. Kurin, N.E. Fleshner, J.A. Medin, Mol. Cancer Ther. **7**, 3018–3028 (2008). 10.1158/1535-7163.MCT-08-0335 10.1158/1535-7163.MCT-08-0335 18790782

[35] K. Hanrahan, A. O’Neill, M. Prencipe, J. Bugler, L. Murphy, A. Fabre, M. Puhr, Z. Culig, K. Murphy, R.W. Watson, Mol. Oncol. **11**, 251–265 (2017). 10.1002/1878-0261.12030 10.1002/1878-0261.12030 PMC5527446 28133913

[36] M. Marin-Aguilera, J. Codony-Servat, O. Reig, J.J. Lozano, P.L. Fernandez, M.V. Pereira, N. Jimenez, M. Donovan, P. Puig, L. Mengual, R. Bermudo, A. Font, E. Gallardo, M.J. Ribal, A. Alcaraz, P. Gascon, B. Mellado, Mol. Cancer Ther. **13**, 1270–1284 (2014). 10.1158/1535-7163.MCT-13-0775 10.1158/1535-7163.MCT-13-0775 24659820

[37] K. Sivasubramaniyan, A. Harichandan, K. Schilbach, A.F. Mack, J. Bedke, A. Stenzl, L. Kanz, G. Niederfellner, H.J. Buhring, Glycobiology **25**, 902–917 (2015). 10.1093/glycob/cwv032 10.1093/glycob/cwv032 PMC4565992 25978997

[38] M. Teodorczyk, S. Kleber, D. Wollny, J.P. Sefrin, B. Aykut, A. Mateos, P. Herhaus, I. Sancho-Martinez, O. Hill, C. Gieffers, J. Sykora, W. Weichert, C. Eisen, A. Trumpp, M.R. Sprick, F. Bergmann, T. Welsch, A. Martin-Villalba, Cell Death Differ. **22**, 1192–1202 (2015). 10.1038/cdd.2014.217 10.1038/cdd.2014.217 PMC4572867 25613377

[39] T.Z. Tan, Q.H. Miow, Y. Miki, T. Noda, S. Mori, R.Y. Huang, J.P. Thiery, EMBO Mol. Med. **6**, 1279–1293 (2014). 10.15252/emmm.201404208 10.15252/emmm.201404208 PMC4287932 25214461

[40] R.L. Siegel, K.D. Miller, H.E. Fuchs, A. Jemal, CA Cancer J. Clin. **72**, 7–33 (2022). 10.3322/caac.21708 10.3322/caac.21708 35020204

[41] T. Karantanos, C.P. Evans, B. Tombal, T.C. Thompson, R. Montironi, W.B. Isaacs, Eur. Urol. **67**, 470–479 (2015). 10.1016/j.eururo.2014.09.049 10.1016/j.eururo.2014.09.049 PMC5301306 25306226

[42] I.F. Tannock, R. de Wit, W.R. Berry, J. Horti, A. Pluzanska, K.N. Chi, S. Oudard, C. Theodore, N.D. James, I. Turesson, M.A. Rosenthal, M.A. Eisenberger, N. Engl. J. Med. **351**, 1502–1512 (2004). 10.1056/NEJMoa040720 10.1056/NEJMoa040720 15470213

[43] D.P. Petrylak, C.M. Tangen, M.H. Hussain, L. P.n. Jr, J.A. Jones, M.E. Taplin, P.A. Burch, D. Berry, C. Moinpour, M. Kohli, M.C. Benson, E.J. Small, D. Raghavan, E.D. Crawford, N. Engl. J. Med. 10.1056/NEJMoa041318 10.1056/NEJMoa041318 15470214

[44] C.E. Kyriakopoulos, Y.H. Chen, M.A. Carducci, G. Liu, D.F. Jarrard, N.M. Hahn, D.H. Shevrin, R. Dreicer, M. Hussain, M. Eisenberger, M. Kohli, E.R. Plimack, N.J. Vogelzang, J. Picus, M.M. Cooney, J.A. Garcia, R.S. DiPaola, C.J. Sweeney, J. Clin. Oncol. **36**, 1080–1087 (2018). 10.1200/JCO.2017.75.3657 10.1200/JCO.2017.75.3657 PMC5891129 29384722

[45] C.J. Sweeney, Y.H. Chen, M. Carducci, G. Liu, D.F. Jarrard, M. Eisenberger, Y.N. Wong, N. Hahn, M. Kohli, M.M. Cooney, R. Dreicer, N.J. Vogelzang, J. Picus, D. Shevrin, M. Hussain, J.A. Garcia, R.S. DiPaola, N. Engl. J. Med. **373**, 737–746 (2015). 10.1056/NEJMoa1503747 10.1056/NEJMoa1503747 PMC4562797 26244877

[46] N.D. James, J.S. de Bono, M.R. Spears, N.W. Clarke, M.D. Mason, D.P. Dearnaley, A.W.S. Ritchie, C.L. Amos, C. Gilson, R.J. Jones, D. Matheson, R. Millman, G. Attard, S. Chowdhury, W.R. Cross, S. Gillessen, C.C. Parker, J.M. Russell, D.R. Berthold, C. Brawley, F. Adab, S. Aung, A.J. Birtle, J. Bowen, S. Brock, P. Chakraborti, C. Ferguson, J. Gale, E. Gray, M. Hingorani, P.J. Hoskin, J.F. Lester, Z.I. Malik, F. McKinna, N. McPhail, J. Money-Kyrle, J. O’Sullivan, O. Parikh, A. Protheroe, A. Robinson, N.N. Srihari, C. Thomas, J. Wagstaff, J. Wylie, A. Zarkar, M.K.B. Parmar, M.R. Sydes, S. Investigators, N. Engl. J. Med. **377**, 338–351 (2017). 10.1056/NEJMoa1702900

[47] B. Bumbaca, W. Li, Acta Pharm. Sin. B **8**, 518–529 (2018). 10.1016/j.apsb.2018.04.007 10.1016/j.apsb.2018.04.007 PMC6089846 30109177

[48] W. Li, L. Qian, J. Lin, G. Huang, N. Hao, X. Wei, W. Wang, J. Liang, Oncotarget **8**, 65143–65151 (2017). 10.18632/oncotarget.17821 10.18632/oncotarget.17821 PMC5630319 29029419

[49] G.S. Palapattu, C. Wu, C.R. Silvers, H.B. Martin, K. Williams, L. Salamone, T. Bushnell, L.S. Huang, Q. Yang, J. Huang, Prostate **69**, 787–798 (2009). 10.1002/pros.20928 10.1002/pros.20928 19189306

[50] A. Hoshino, B. Costa-Silva, T.L. Shen, G. Rodrigues, A. Hashimoto, M. Tesic Mark, H. Molina, S. Kohsaka, A. Di Giannatale, S. Ceder, S. Singh, C. Williams, N. Soplop, K. Uryu, L. Pharmer, T. King, L. Bojmar, A.E. Davies, Y. Ararso, T. Zhang, H. Zhang, J. Hernandez, J.M. Weiss, V.D. Dumont-Cole, K. Kramer, L.H. Wexler, A. Narendran, G.K. Schwartz, J.H. Healey, P. Sandstrom, K.J. Labori, E.H. Kure, P.M. Grandgenett, M.A. Hollingsworth, M. de Sousa, S. Kaur, M. Jain, K. Mallya, S.K. Batra, W.R. Jarnagin, M.S. Brady, O. Fodstad, V. Muller, K. Pantel, A.J. Minn, M.J. Bissell, B.A. Garcia, Y. Kang, V.K. Rajasekhar, C.M. Ghajar, I. Matei, H. Peinado, J. Bromberg, D. Lyden, Nature **527**, 329–335 (2015). 10.1038/nature15756 10.1038/nature15756 PMC4788391 26524530

[51] J. Li, N. Gao, Z. Gao, W. Liu, B. Pang, X. Dong, Y. Li, T. Fan, Front. Cell Dev. Biol. **9**, 737962 (2021). 10.3389/fcell.2021.737962 10.3389/fcell.2021.737962 PMC8581179 34778252

[52] N. Milman, L. Ginini, Z. Gil, Drug. Resist. Updat. **45**, 1–12 (2019). 10.1016/j.drup.2019.07.003 10.1016/j.drup.2019.07.003 31369918

[53] A. Kumar, P. Kumar, M. Sharma, S. Kim, S. Singh, S.J. Kridel, G. Deep, Cancer Drug. Resist. **5**, 612–624 (2022). 10.20517/cdr.2022.26 10.20517/cdr.2022.26 PMC9511801 36176762

[54] C. Corcoran, S. Rani, K. O’Brien, A. O’Neill, M. Prencipe, R. Sheikh, G. Webb, R. McDermott, W. Watson, J. Crown, L. O’Driscoll, PLoS One **7**, e50999 (2012). 10.1371/journal.pone.0050999 10.1371/journal.pone.0050999 PMC3519481 23251413

[55] S. Jorfi, E.A. Ansa-Addo, S. Kholia, D. Stratton, S. Valley, S. Lange, J. Inal, Sci. Rep. **5**, 13006 (2015). 10.1038/srep13006 10.1038/srep13006 PMC4548233 26302712

[56] J. Jiang, J. Li, X. Zhou, X. Zhao, B. Huang, Y. Qin, Front. Oncol. **12**, 864980 (2022). 10.3389/fonc.2022.864980 10.3389/fonc.2022.864980 PMC8964004 35359397

[57] W. Zhang, M.L. Ding, J.N. Zhang, J.R. Qiu, Y.H. Shen, X.Y. Ding, L.F. Deng, W.B. Zhang, J. Zhu, Sci. Rep. **5**, 9604 (2015). 10.1038/srep09604 10.1038/srep09604 PMC4389812 25853231

[58] A. Aloia, E. Petrova, S. Tomiuk, U. Bissels, O. Deas, M. Saini, F.M. Zickgraf, S. Wagner, S. Spaich, M. Sutterlin, A. Schneeweiss, M. Reitberger, S. Ruberg, B. Gerstmayer, D. Agorku, S. Knobel, A. Terranegra, M. Falleni, L. Soldati, M.R. Sprick, A. Trumpp, J.G. Judde, A. Bosio, S. Cairo, O. Hardt, Breast Cancer Res. **17**, 146 (2015). 10.1186/s13058-015-0652-6 10.1186/s13058-015-0652-6 PMC4660783 26607327

[59] Y.W. Lou, P.Y. Wang, S.C. Yeh, P.K. Chuang, S.T. Li, C.Y. Wu, K.H. Khoo, M. Hsiao, T.L. Hsu, C.H. Wong, Proc. Natl. Acad. Sci. U. S. A. **111**, 2482–2487 (2014). 10.1073/pnas.1400283111 10.1073/pnas.1400283111 PMC3932869 24550271

[60] Z. Noto, T. Yoshida, M. Okabe, C. Koike, M. Fathy, H. Tsuno, K. Tomihara, N. Arai, M. Noguchi, T. Nikaido, Oral Oncol **49**, 787–795 (2013). 10.1016/j.oraloncology.2013.04.012 10.1016/j.oraloncology.2013.04.012 23768762

[61] J. Cheng, K. Yang, Q. Zhang, Y. Yu, Q. Meng, N. Mo, Y. Zhou, X. Yi, C. Ma, A. Lei, Y. Liu, Sci. Rep. **6**, 16993 (2016). 10.1038/srep16993 10.1038/srep16993 PMC4726385 26787499

[62] Y. Nakamura, Y. Miyata, T. Matsuo, Y. Shida, T. Hakariya, K. Ohba, T. Taima, A. Ito, T. Suda, S.I. Hakomori, S. Saito, H. Sakai, Glycoconjugate J. **36**, 409–418 (2019). 10.1007/s10719-019-09882-2 10.1007/s10719-019-09882-2 PMC6744380 31243630

[63] R. Liu, Y. Chen, G. Liu, C. Li, Y. Song, Z. Cao, W. Li, J. Hu, C. Lu, Y. Liu, Cell Death Dis. **11**, 797 (2020). 10.1038/s41419-020-02998-6 10.1038/s41419-020-02998-6 PMC7515865 32973135

[64] F. Liu, K. Bardhan, D. Yang, M. Thangaraju, V. Ganapathy, J.L. Waller, G.B. Liles, J.R. Lee, K. Liu, J. Bio.l Chem. **287**, 25530–25540 (2012). 10.1074/jbc.M112.356279 10.1074/jbc.M112.356279 PMC3408167 22669972

[65] Y. Du, X. Teng, N. Wang, X. Zhang, J. Chen, P. Ding, Q. Qiao, Q. Wang, L. Zhang, C. Yang, Z. Yang, Y. Chu, X. Du, X. Zhou, W. Hu, J. Bio.l Chem. **289**, 2711–2724 (2014). 10.1074/jbc.M113.525501 10.1074/jbc.M113.525501 PMC3908404 24338025

[66] M. Le Gallo, A. Poissonnier, P. Blanco, P. Legembre, Front. Immunol. **8**, 1216 (2017). 10.3389/fimmu.2017.01216 10.3389/fimmu.2017.01216 PMC5623854 29021794

[67] T. Szarvas, S. Sevcenco, O. Modos, D. Keresztes, P. Nyirady, A. Csizmarik, R. Ristl, M. Puhr, M.J. Hoffmann, C. Niedworok, B. Hadaschik, A. Maj-Hes, S.F. Shariat, G. Kramer, BJU Int. **122**, 695–704 (2018). 10.1111/bju.14415 10.1111/bju.14415 29802777

[68] C. Xu, M. Jung, M. Burkhardt, C. Stephan, D. Schnorr, S. Loening, K. Jung, M. Dietel, G. Kristiansen, Prostate **62**, 224–232 (2005). 10.1002/pros.20134 10.1002/pros.20134 15389793

