# Supplementary material for: Pre-existing cell subpopulations in primary prostate cancer tumors display surface fingerprints of docetaxel-resistant cells
Source: Cell Oncol (Dordr). 2024 Aug 20;48(1):205–18. doi: 10.1007/s13402-024-00982-2 (PMC11850551; doi:10.1007/s13402-024-00982-2)

Supplementary Figure S1

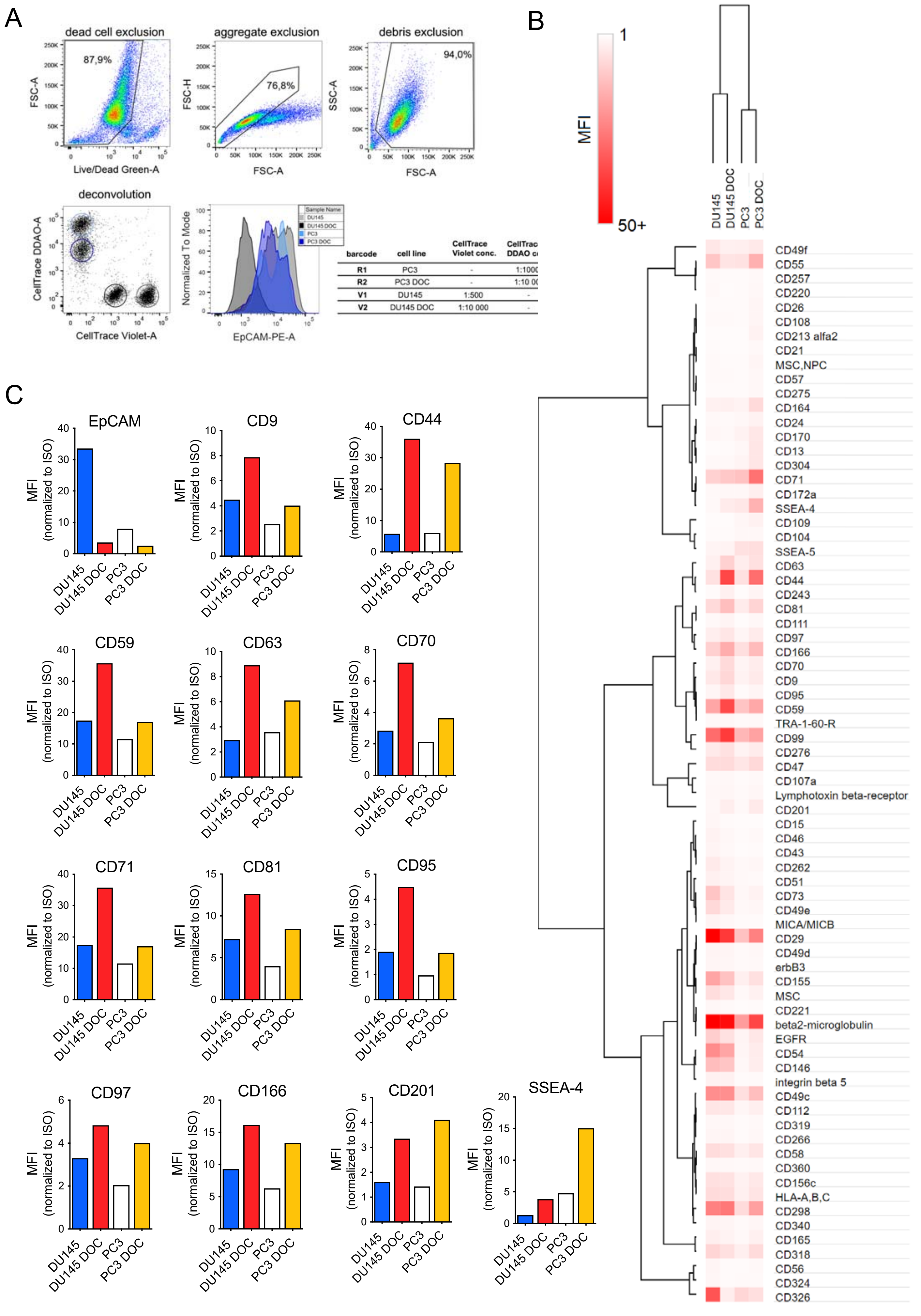

Supplementary Figure S2

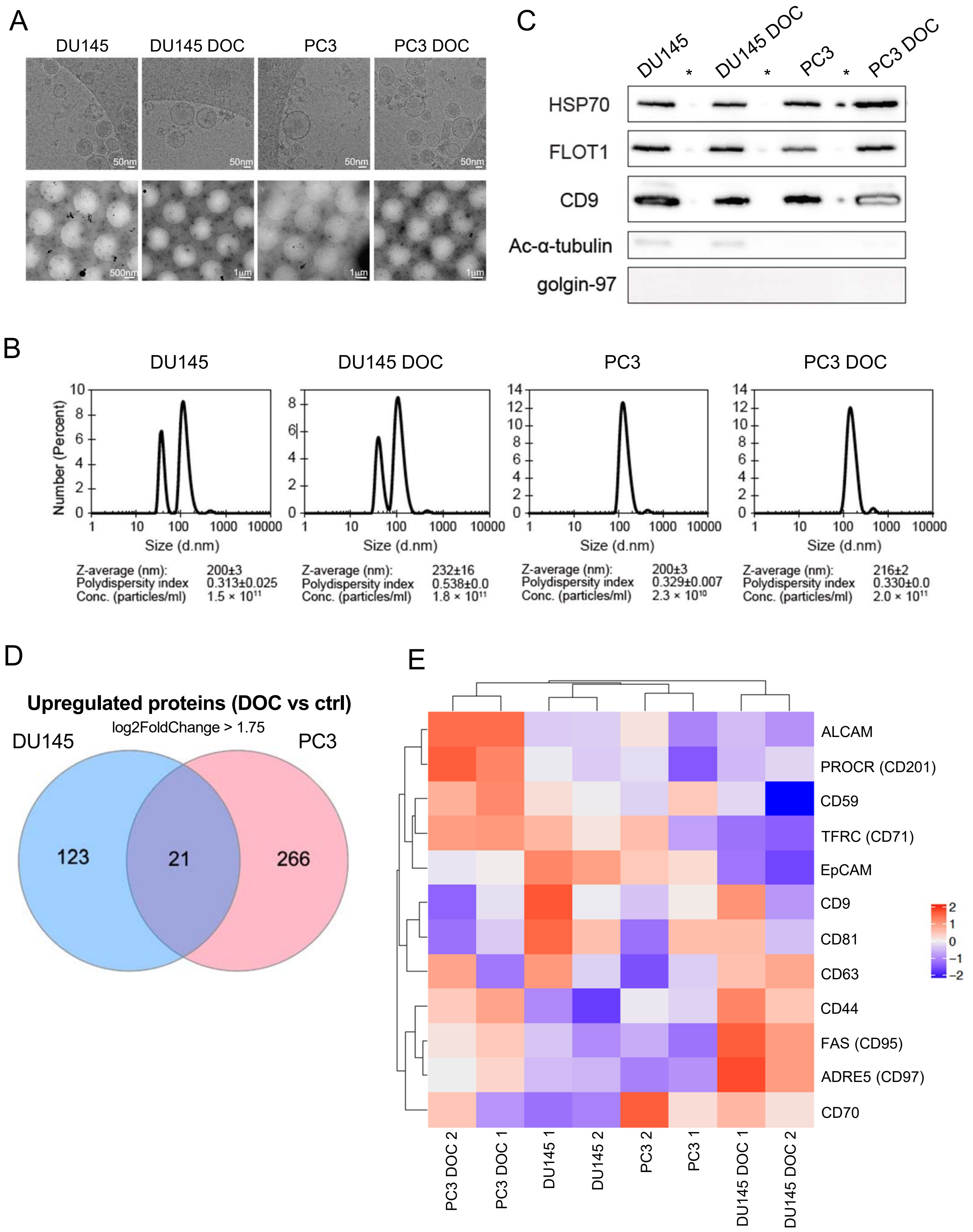

Supplementary Figure S3

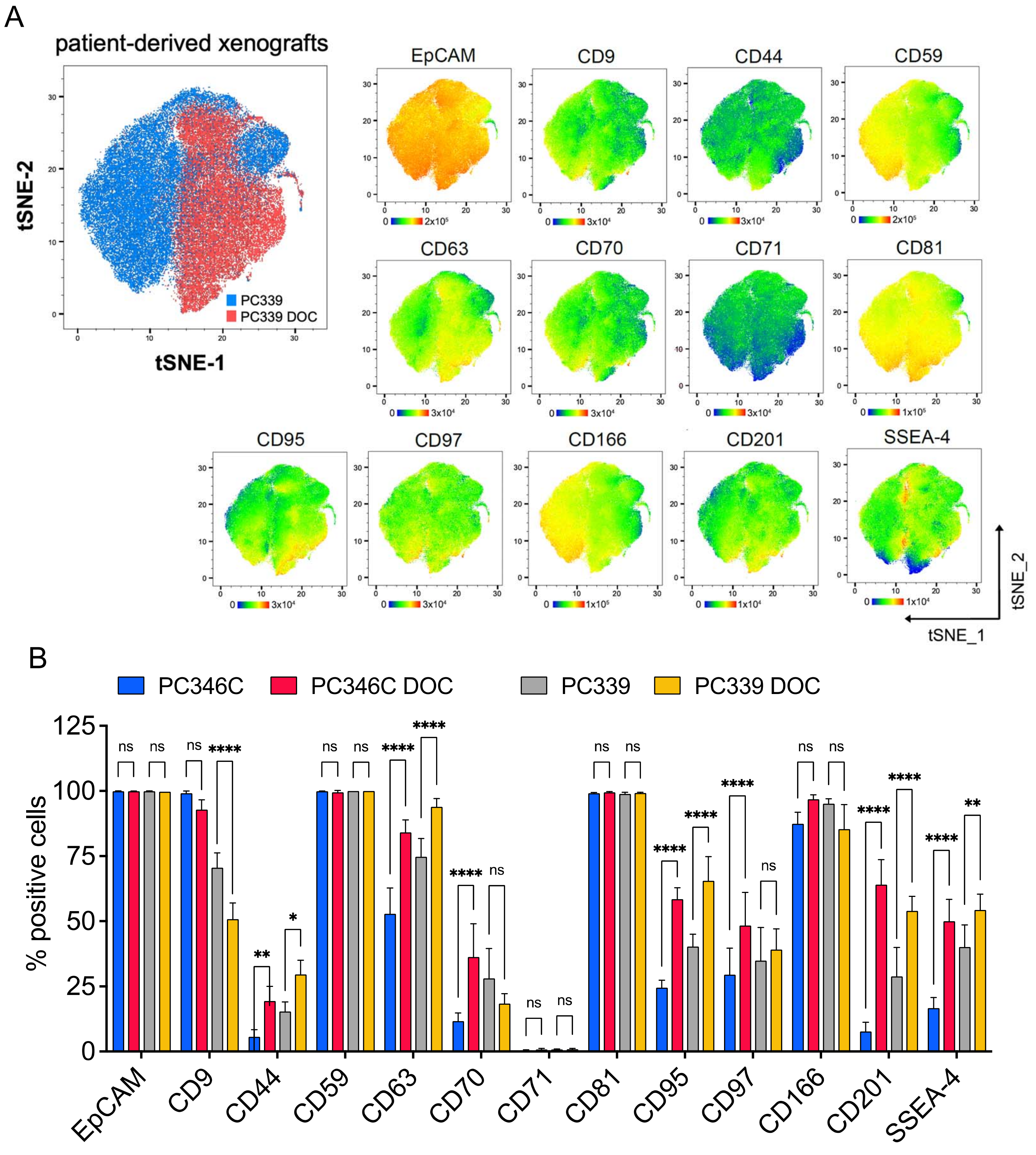

Supplementary Figure S4

A

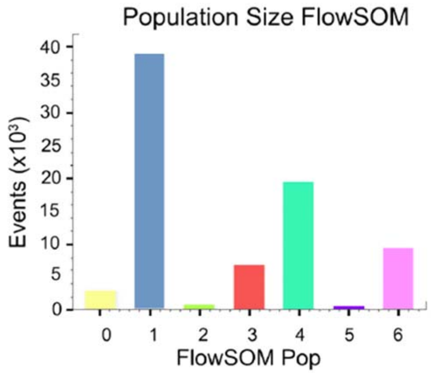

B

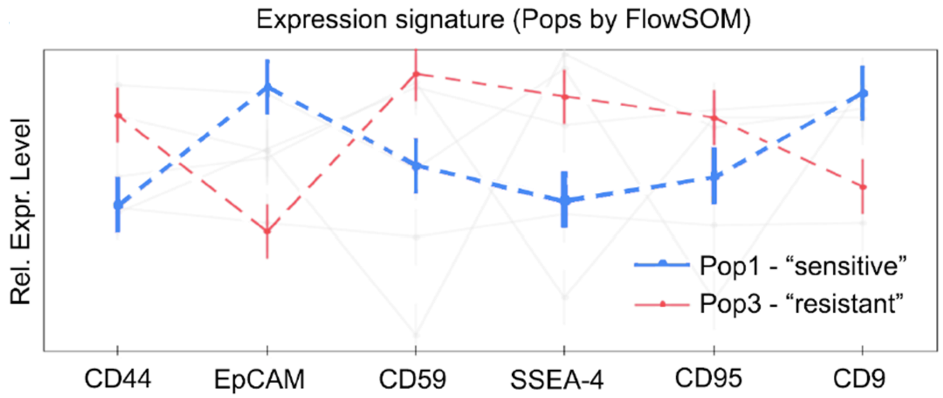

Supplementary Figure S5

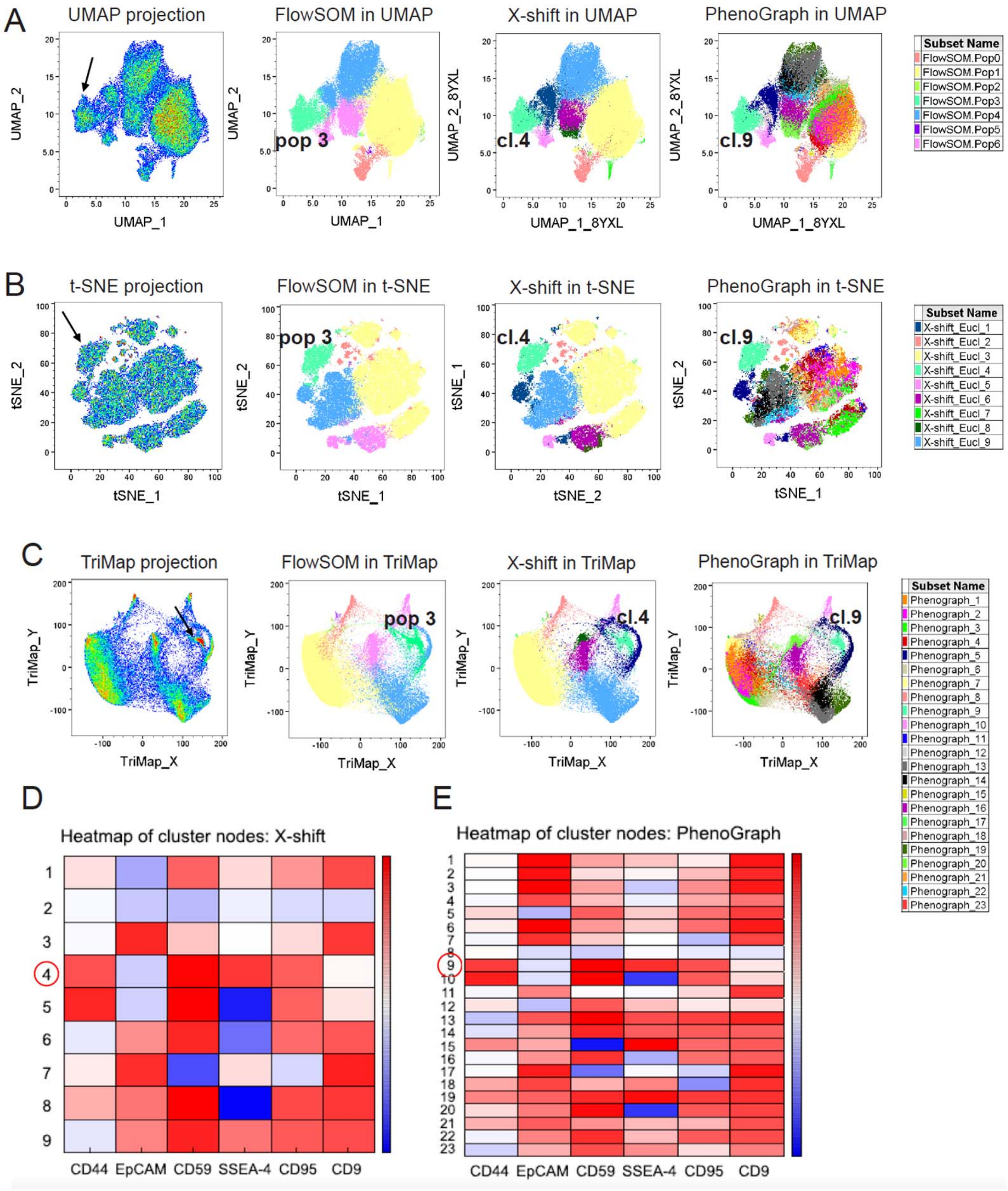

Supplementary Figure S6

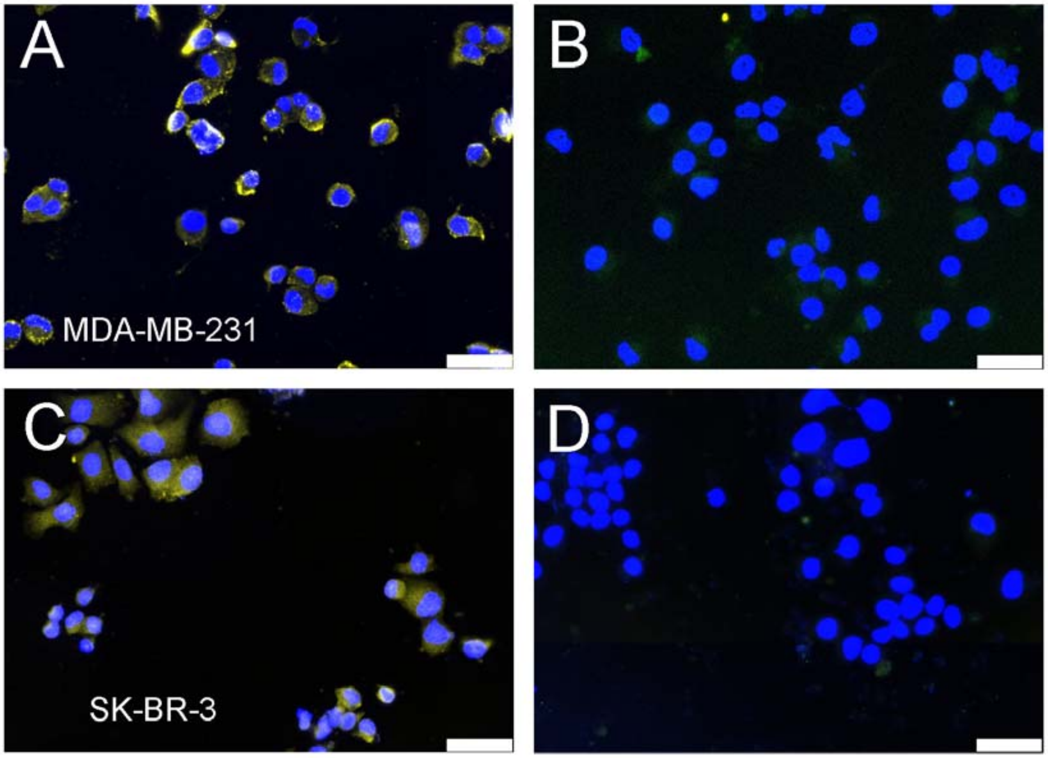

Supplementary Figure S7

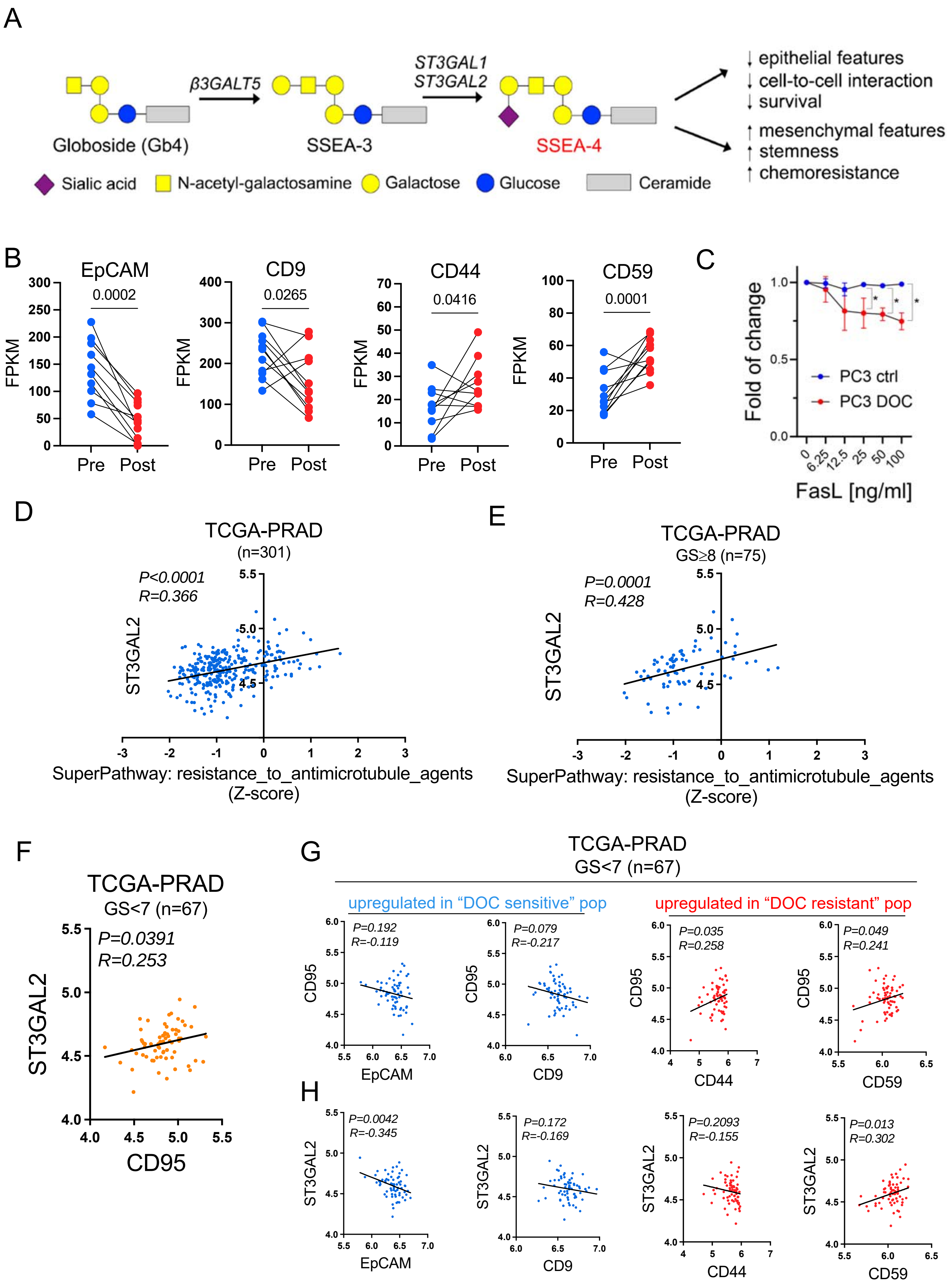

Supplement: Supplementary file 1 — Supplementary Material 1 [file 13402_2024_982_MOESM1_ESM.pdf]
