# Supplementary material for: Pre-existing cell subpopulations in primary prostate cancer tumors display surface fingerprints of docetaxel-resistant cells
Source: Cell Oncol (Dordr). 2024 Aug 20;48(1):205–18. doi: 10.1007/s13402-024-00982-2 (PMC11850551; doi:10.1007/s13402-024-00982-2)
Supplement: Supplementary file 2 — Supplementary Material 2 [file 13402_2024_982_MOESM2_ESM.docx]

**Supplementary Information for:**

Drápela, S. *et al.*, Pre-existing cell subpopulations in primary prostate cancer tumors display surface fingerprint of docetaxel-resistant cells

**Supplementary Material and Methods**

**Cell lines, xenografts, and chemicals**

Docetaxel-resistant (DOC) DU145 and PC3 prostate cancer cell lines were derived as previously reported.[[1](#_ENREF_1)] Docetaxel resistance was maintained by a continuous supply of docetaxel (cat. no. 9886, Cell Signaling, Danvers, MA, USA) in the final concentration of 12.5 nM. Cell lines were maintained at 37°C (5% CO_2_) in RPMI 1640 media (cat. no. 72400-021, Thermo Fisher Scientific (TFS), Waltham, MA, USA) supplemented with 10% fetal bovine serum (cat. no. 10270106, TFS, USA) and 100 U/mL penicillin/streptomycin (cat.no. XC-A4122/100, Biosera, France). The PDXs were established and cultured as described previously [[2](#_ENREF_2), [3](#_ENREF_3)]. Docetaxel resistance was maintained by a continuous supply of docetaxel in the final concentration of 1 nM. PDXs were maintained at 37°C (5% CO_2_) in DMEM/F12 1:1 media (cat. no. 31330, TFS) supplemented with 2 % fetal bovine serum and 100 U/mL penicillin/streptomycin, 0.01% BSA (cat. no. 11930, Serva, Germany), 10 ng/mL rhEGF (cat.no. E9644, Merck, Germany), 1 % ITS-G (cat. no. 41400-045, TFS, USA), 0.5 ug/mL hydrocortisone (cat.no. H0888, Merck, Germany), 1 nM triiodothyronine (cat.no. IRMM469, Merck, Germany), 0.1 mM O-phosphorylethanolamine (cat.no. P0503, Merck, Germany), 50 ng/mL cholera toxin (cat.no. C8052, Merck, Germany), 0.1 µg/mL fibronectin from human plasma (cat.no. F0895, Merck, Germany), 20 µg/mL fetuin from fetal bovine serum (cat.no. F3385, Merck, Germany) and 0.1 nM methyltrienolone (R1881) (cat. no. 816439, MolPort, Latvia), recombinant human FasL (CD95L) and its enhancer (ALX-850-014-K102, Alexis Biochemicals, USA), CyQuant NF Cell Proliferation Assay kit (C35006, Invitrogen, TFS).

**Antibody-based cell surface screening**

For surface profiling, cell lines were expanded in appropriate medium and harvested by incubation in 1.35 mM EDTA solution in PBS to allow non-enzymatic weakening of the cell junctions (2 mins for DU145 and DU145 DOC and 3 mins for PC3 and PC3 DOC cells) followed by mild HyClone HyQTase (cat.no. SV3003001, GE Healthcare Bio-Sciences AB, Sweden) incubation for 5 mins, as some antigens included in the screen are sensitive to massive trypsinization and differences in cell harvesting would obscure the results. HyClone HyQTase was then neutralized with a cell culture medium containing 10% FBS (TFS, USA). Suspension of each cell line was counted, washed with PBS, and 5x10^7^ cells were barcoded with CellTrace Violet or/and CellTrace DDAO (Far Red, TFS, USA) amine-reactive fluorescent dyes diluted in PBS (1 mL staining solution per 1x10^7^ cells), for 15 min at 37°C while gently shaking on the vertical rotator. The remaining unreacted dye was then quenched by adding a cell culture medium containing 10% FBS and incubating for 30 min at 37°C in the dark. Cell suspensions were then washed with PBS and stained with LIVE/DEAD Green Fixable Dead Cell Stain diluted 1:1000 in PBS for 15 min at 4°C (1 mL staining solution per 1x10^7^ cells; TFS, USA). The cells were washed with PBS, filtered via 100 μm cell strainer to remove large cell aggregates, re-counted, and 45x10^7^ cells from each cell line were then pooled in 27 mL of Cell Stain buffer (part of LEGENDScreen Kit, cat. no. 700001; Biolegend, San Diego, CA, USA) and filtered via 70 μm cell strainer. Cell suspension (75 μL/well, equal to a pool of 0.75x10^6^ cells/well) was then dispensed into reconstituted LEGENDScreen Human Cell Screening PE Kit 96 well plates and incubated for 20 min at 4°C in the dark. After reconstitution with MQ water, each well contained 25 μL of single, validated, and pre-titrated antibody conjugated with Phycoerythrin (PE). Following the staining, the plates were spun down 500g for 6 min, and the supernatant was dumped by quickly inverting and flicking the plate. The cells were washed with a Cell Stain Buffer and fixed in a Fixation Buffer (part of LEGENDScreen Kit) for 10 min at RT as recommended by the manufacturer. The cells were washed twice, resuspended in Cell Stain Buffer, and processed for analysis.

**Preparation of vesicle-free medium for cell culture**

Vesicle-free medium is prepared as described in the Current Protocol in Cell Biology [[4](#_ENREF_4)]. In brief, RPMI1640 medium was prepared as "5x concentrated" by supplementing 50 % FBS, 250 units/ml penicillin, and 2500 units/ml streptomycin (P/S). The "5x concentrated" medium was ultracentrifuged at 100,000 x g, 4 °C in a SW32Ti rotor for 5 h. The supernatant was then diluted with RPMI1640 medium to reach the 10% FBS, 50 units/ml penicillin, and 50 units/ml streptomycin concentrations, and filter sterilized using 0.22 μm vacuum-driven filter in the hood.

**Cell culture for condition media (CM) production/EVs isolation**

Cells were expanded in standard culture medium (RPMI1640 + 25mM HEPES (+stable glutamine) supplemented by 10% FBS, + P/S). To isolate the EVs, cells were harvested by 0.05% trypsin/EDTA, washed twice in PBS, and plated onto new plates in a vesicle-free medium. The speed of growth in the vesicle-free medium was determined experimentally ahead for each cell line, and cells were plated at the confluence, leading to their 90% confluency at the time of CM harvest to maximize the amount of produced EVs. Twelve 150mm dishes (each with 20 ml of vesicle-free media) were used for one batch of EV isolates for each cell line. Supernatants were collected 72 hours later.

**Harvesting the CM**

CM was collected from plates and spun at 1,500 x g for 10 min at 10°C (to remove cell debris and apoptotic bodies), and the supernatant was used for EV isolation. Cells were counted, and their viability was assessed; only CM harvested from plates with cells above 90% viability was used to isolate EVs.

**Ultracentrifugation with sucrose cushion**

As described previously, EVs were isolated by differential ultracentrifugation coupled to sucrose cushion flotation step [[5](#_ENREF_5), [6](#_ENREF_6)]. In short, CM was centrifuged in 38.5-ml Open-top UC tubes using SW32Ti rotor in Optima XPN 90k ultracentrifuge (all Beckman Coulter). Firstly, 12,000 × g for 20 min at 10°C to remove large vesicles and apoptotic bodies. The supernatant was subsequently ultracentrifuged at 100,000 × g for 2 h 20 min at 10°C. Pellet was resuspended in filtered PBS (fPBS; 0.22 μm PVDF filter) and carefully layered onto 4 ml of the sucrose cushion (30% sucrose in 20 mM Tris pH 7.6 in D2O, filter sterilized) in a new UC tube and ultracentrifuged at 100,000 × g, for 1 h 10 min at 10°C. Approximately 6 ml of fPBS and the cushion at the interface of both layers was collected, transferred into a new UC tube, filled with fPBS, and ultracentrifuged at 100,000 × g, overnight (O/N) (approx. 16h) at 10°C. The pellet of EVs was resuspended in 100 μl of fPBS.

**Cryo-electron microscopy (cryo-EM)**

3.5 μl of EV sample per grid was used; samples were vitrified using FEI vitrobot Mark IV on Cu Quantifoil 2/1 mesh 300 grids. The following settings were used: blot force -4; blot time 6 s; wait time 120 s. Data was acquired on the TalosArctica cryo-TEM instrument (TFS, USA) at the Cryo-Electron Microscopy and Tomography Core Facility (CEITEC, Brno, CZ).

**Dynamic Light Scattering**

The size and concentration of EVs was assessed with the multi-angled dynamic light scattering technique (MADLS®). 50 µl of the sample suspension was pipetted into low-volume quartz batch cuvette ZEN2112 (Malvern Panalytical Ltd, UK) and measured using Zetasizer Ultra (Malvern Panalytical Ltd, UK) equipped with HeNe Laser (633 nm) and three detection angles (173°, 90°, and 13°) at a constant temperature of 25°C. ZS Xplorer software version 1.5.0.163 (Malvern Panalytical Ltd, UK) was used to evaluate the obtained data; the results are reported as mean value (n = 3) ± standard deviation.

**SDS-PAGE and Western Blotting**

Lysates of EVs were prepared by direct lysis in 5x concentrated reducing Laemmli buffer. Lysates were separated on 15% SDS-PAGE and transferred to Immobilon-P Membrane (Millipore). After blocking of non-specific binding in 5% non-fat milk in PBS with 0.5% Tween 20 membranes were incubated in primary antibodies mixtures O/N at 4 °C on a rocker. Following extensive washing in PBS with 0.5% Tween 20, membranes were incubated with secondary antibodies for 1 h at RT. After extensive final washing, membranes were developed by chemiluminescent ECL solutions Immobilon Western (Millipore), and the signal was detected using Fusion SL (Vilber Lourmat). The following antibodies were used: mouse anti-Hsp70 (sc-24, SCBT, dilution 1:1,000), rabbit anti-Flotillin1 (A3023, ABclonal, dilution 1:500), rabbit anti-CD9 (A19027, ABclonal, dilution 1:500), Acetyl-α-Tubulin (Lys40) (CS-5335, Cell Signaling, dilution 1:1,000) and golgin-97 (A-21270, Invitrogen, dilution 1:500).

**Samples preparation for LC-MS analyses**

EV suspension was solubilized and lysed in a solubilization buffer (5% SDS, 250mM DTT, 250mM Tris-HCl pH 7.6). The lysates were used for filter-aided sample preparation (FASP, 30kDa cut-off cartridges) as described elsewhere [[4](#_ENREF_4)] using 0.5 μg of trypsin (sequencing grade; Promega). The resulting peptides were extracted into LC-MS vials with 2.5% formic acid (FA) in 50% acetonitrile (ACN) and 100% ACN with the addition of polyethylene glycol (20,000; final concentration 0.001%) [[7](#_ENREF_7)], and concentrated in a SpeedVac concentrator (TFS, USA), followed by these peptides were taken for LC-MS analysis. Four biological replicates were analyzed, with two being excluded due to quality control issues.

**LC-MS analysis of tryptic peptides**

LC-MS/MS analyses of all peptide mixtures were done using the Ultimate 3000 RSLCnano system connected to the Orbitrap Exploris 480 mass spectrometer (TFS). Before LC separation, tryptic digests were online concentrated and desalted using a trapping column (300 μm × 5 mm, μPrecolumn, 5μm particles, Acclaim PepMap100 C18, TFS, USA; temperature of 40 ºC). After washing the trapping column with 0.1% formic acid (FA), the peptides were eluted (flow rate - 300 nl/min) from the trapping column onto an analytical column (EASY spray column, Acclaim Pepmap100 C18, 2 µm particles, 75 μm × 250 mm; TFS, USA) by 90 min linear gradient program (5-37% of mobile phase B; mobile phase A: 0.1% FA in water; mobile phase B: 0.1% FA in 80% ACN). The trapping and analytical columns were equilibrated before sample injection into the sample loop. The analytical column was installed in the EASY-Spray ion source (TFS, USA) according to the manufacturer's instructions with a column temperature of 40 ºC. Spray voltage and sheath gas were set to 1.9kV and 1, respectively. Data were acquired in a data-independent acquisition mode (DIA). The survey scan covered an m/z range of 350-1400 at a resolution of 60,000 (at m/z 200) and a maximum injection time of 55 ms. HCD MS/MS (27% relative fragmentation energy) were acquired in the m/z range of 200-2000 at 30,000 resolution (maximum injection time 55 ms). Overlapping windows scheme in m/z range from 400 to 800 were used as isolation window placements – see transitions_list.xlsx file for more details. DIA data were processed in DIA-NN [[8](#_ENREF_8)] (version 1.8) in library free mode against modified cRAP database (based on http://www.thegpm.org/crap/; 111 sequences in total) and UniProtKB protein database for Homo sapiens (htttps://ftp.uniprot.org/pub/databases/uniprot/current_release/knowledgebase/reference_proteomes/Eukaryota/UP000005640/UP000005640_9606.fasta.gz; version 2022/03, number of protein sequences: 20,577). No optional modification, carbamidomethylation as fixed modification and trypsin/P enzyme with 1 allowed missed cleavages, and peptide lengths of 7-30 were set during the library preparation. False discovery rate (FDR) control was set to 1% FDR. MS1 and MS2 accuracies and scan window parameters were set based on the initial test searches (median value from all samples ascertained parameter values). MBR was switched on.

**Xenograft mouse experiments and patient-derived xenograft tumor processing**

Floating spheroids of PDXs cell cultures were harvested, washed with 1.35 mM EDTA solution in PBS, and subjected to non-enzymatic weakening of the cell junctions using HyClone HyQTase for 10 min at 37 °C. HyClone HyQTase was then neutralized with a cell culture medium containing 10% FBS. The suspension of each cell model was filtered via 70 μm cell strainer to remove large cell aggregates, counted using CASY cell counter (OLS OMNI Life Science, Germany) and a total of 1×10^6^ of PDX cells were resuspended in the 1:1 mix of ice‐cold PBS and Matrigel (Corning Incorporated, NY, USA) and inoculated subcutaneously into the right flank (dorsally) of six‐week‐old male SHO mice. The experimental unit refers to a single animal. Mice were housed in sterile cages in a temperature-controlled room with a 12-h light–dark cycle. No criteria for including and excluding animals during the experiments were applied. The confounders were not controlled. The tumors were surgically excised and enzymatically dissociated using digestion media containing 2 mg/mL Collagenase Type I (cat.no. LS 004194, Worthington, Lakewood, NJ, USA) and 0.6 U/mL Dispase II (Roche, Switzerland) in RPMI 1640 for 1 hour at 37°C on a horizontal rotator. Samples were then treated with 15 μg/mL DNase I (Roche, Switzerland) for 5 min at 37°C, washed with sterile PBS, and filtered through 100 μm and 50 μm cell strainers to remove large cell aggregates. After washing, red blood cells were lysed with ACK buffer (155 mM ammonium chloride, 10 mM potassium bicarbonate, and 100 μM EDTA solution in sterile MQ water). Cells were counted using the CASY TT cell counter, and 1,5×10^6^ cells were used to stain viability and surface marker expression. The animal protocol was prepared and approved before the experiment was started. All European Union Animal Welfare lines (EU Directive 2010/63/EU for animal experiments) were respected. Animal experiments were approved by the Ethical Committee of IBP CAS and REKOZ, Academy of Sciences of the Czech Republic (AVCR 65/2016), supervised by the local ethical committee, and performed by certified individuals (SD and KS).

**Prostate cancer tissue processing**

Tissue samples were minced into 1 mm pieces and enzymatically digested in HBSS buffer (Hank's Balanced Salt Solution + 0,035% NaHCO_3_) containing 2.5 mg/mL Collagenase type I and 10 μM Y-27632 dihydrochloride (cat.no. sc-281642A, Santa Cruz Biotechnology, Dallas, TX, USA), for 2-4 hours (depending on tissue toughness) at 37°C. Gentle agitation (40 RPM) was used to prevent viable cell loss and non-specific surface epitope cleavage. Samples were then treated with 15 μg/mL DNase I for 5 min at 37°C, washed with sterile PBS, filtered through a 50 μm cell strainer, and subjected to staining in fresh condition. Patient characteristics are provided in Table S3

**Spectral flow cytometry analysis**

For multicolor panel testing, the single-cell suspensions from PDXs and patient samples were washed with PBS and subjected to red blood cell lysis with ACK buffer, followed by filtration via a 50 μm strainer. All suspensions were further stained under non-sterile conditions with LIVE/DEAD Yellow Fixable Dead Cell Stain diluted 1:500 in PBS for 15 min at RT (100 μL staining solution per 1x10^6^ cells; TFS, USA) to exclude dead cells from the analysis. Next, for PDXs, a cocktail of fluorochrome-conjugated primary antibodies containing anti-human CD298, CD9, CD44, CD59, CD63, CD70, CD71, CD81, CD95, CD97, CD166 (ALCAM), CD201, SSEA-4, EpCAM was used for extracellular staining in a single tube. For the patient samples, the same panel of antibodies (excluding CD298) was enriched by FITC-conjugated anti-human CD45, CD31, and CD90 antibodies. Suspensions were stained in sodium azide (NaN_3_) buffer (0.02% sodium azide + 1% BSA in PBS) enriched by Super Bright Complete Staining Buffer (cat.no. SB-4401-42, TFS, USA) for 20 min at 4 °C, then washed and processed to analysis.

**Immunofluorescence staining of SSEA4**

Cryosections or frozen cells (MDA-MB-231 and SK-BR-3) were blocked with an Anti-hu Fc receptor binding inhibitor (ThermoFisher Scientific, USA) for 20 min at room temperature. After incubation with the mouse monoclonal antibody against SSEA4 (clone MC13-70, DSHB, USA) for 60 minutes at 4 ⁰C, the cryosections were washed three times with PBS-T solution and incubated with the secondary antibody AlexaFluor®555 Conjugate (Cell Signaling, USA) for 30 minutes at 4 ⁰C, protected from light. Antibodies were diluted in Flow cytometry staining buffer (ThermoFisher, USA). After washing with PBS-T, DAPI (Serva, Germany) was added for 10 minutes at room temperature, and the cover glass was mounted using a drop of Mowiol (Merck, USA). The slides were imaged with Slideview VS200 microscope (Olympus, Japan).

Staining of SSEA4 was also tested on formalin-fixed paraffin-embedded cells as well as on cells fixed directly on a glass slide. However, these pretreatments led to unspecific signals in SK-BR-3 cells without SSEA4 expression (see Figure S6). Therefore, we adopted the "flow-cytometry" staining protocol for frozen tissues (and frozen cells) as described above.

**Immunohistochemistry**

Immunostaining of formalin-fixed paraffin-embedded tissues with appropriate antibodies, E-cadherin, clone NCH-38, Dako, 1:50, Ventana Ultra, Roche, N-cadherin, clone 6G11, Dako, 1:50, antigen retrieval EnVision Flex, Dako and CD95, GM30, Novocastra, 1:50, antigen retrieval EnVision Flex enhanced, Dako was done according to standard techniques. Protein expression was assessed semiquantitatively by an expert prostate cancer pathologist using the histoscore method, where the percentage of positive cells (0–100%) is multiplied by staining intensity (0–3), resulting in a final histoscore that ranges between 0 and 300.

**Data analysis**

As for *Antibody-based cell surface screening*, compensations were calculated automatically in BD FACSuite™ Software (BD) from single-conjugate stained UltraComp eBeads (eBioscience) and applied during data acquisition and analysis. Gates for positivity and isotype median of fluorescence were set based on isotype controls. For median fluorescence index (MFI) calculation, the mean from all isotype controls for each cell line was used. The table showing the percentage of positivity and MFI for each cell line and each analyzed antigen is available in Supplementary Tables S1 and S2. As for *Spectral flow cytometry analyses,* spectral overlaps were calculated and compensated based on the fluorochrome spectra uploaded into the Spectral Library using positive controls (cell lines) and following the automatic spectral unmixing algorithm in SONY SP6800 Software (SONY, Japan). Fluorescence minus one (FMO) controls were measured for all fluorochromes and used for regular gating. For the data visualization using various algorithms listed below, the number of events was downsampled to 1 x 10^4^ per patient sample. Such samples were concatenated and subjected to multidimensional reduction algorithms. We used tSNE algorithm [[9](#_ENREF_9)] for mapping high-dimensional cytometry data onto two dimensions. tSNE plotted individual cells in a visual similar to a scatter plot while using all pairwise distances in high dimension to determine each cell's location in the plot. The tSNE plots are visualized in pseudocolor, and each dot represents a single cell – the lowest expression of the selected marker is in dark blue, and the highest expression is in dark red; the corresponding scale is next to each plot and reflects the marker expression level. Similar to tSNE, the Uniform Manifold Approximation and Projection (UMAP) algorithm, enabling nonlinear dimension reduction, was used to distribute data on the Riemannian manifold uniformly [[10](#_ENREF_10)]. To further visualize multidimensional data in self-organizing maps, we applied the two-level clustering algorithm FlowSOM [[11](#_ENREF_11)]. Briefly, in such visualization, each node represents a cluster of a specific subpopulation of cells, and pie charts of the node reflect the contribution of different markers to the phenotype of the cell cluster. Since FlowSOM enables an adjustable number of clusters for visualization, the K-finder algorithm was applied to selected parameters before FlowSOM analysis to set the "K" value and thus approximate the number of clusters unbiasedly. The dimensionality reduction algorithm TriMap (not published, arXiv:1910.00204) and other clustering algorithms such as PhenoGraph [[12](#_ENREF_12)] and X-shift [[13](#_ENREF_13)] were used to validate the presence of FlowSOM-identified clusters. Only viable, single cells were included in the analyses.

**Supplementary References**

1 M. Puhr, J. Hoefer, G. Schafer, H.H. Erb, S.J. Oh, H. Klocker, I. Heidegger, H. Neuwirt and Z. Culig, Am J Pathol 181, 2188-2201 (2012) doi: 10.1016/j.ajpath.2012.08.011

S0002-9440(12)00653-0 [pii]

2 E.S. de Morree, R. Bottcher, R.J. van Soest, A. Aghai, C.M. de Ridder, A.A. Gibson, R.H. Mathijssen, H. Burger, E.A. Wiemer, A. Sparreboom, R. de Wit and W.M. van Weerden, Br J Cancer 115, 674-681 (2016) doi: 10.1038/bjc.2016.251

bjc2016251 [pii]

3 W.M. van Weerden, C. Bangma and R. de Wit, Br J Cancer 100, 13-18 (2009) doi: 10.1038/sj.bjc.6604822

6604822 [pii]

4 J.R. Wisniewski, A. Zougman, N. Nagaraj and M. Mann, Nat Methods 6, 359-362 (2009) doi: 10.1038/nmeth.1322

5 V. Pospichalova, J. Svoboda, Z. Dave, A. Kotrbova, K. Kaiser, D. Klemova, L. Ilkovics, A. Hampl, I. Crha, E. Jandakova, L. Minar, V. Weinberger and V. Bryja, J Extracell Vesicles 4, 25530 (2015) doi: 10.3402/jev.v4.25530

6 A. Kotrbova, K. Stepka, M. Maska, J.J. Palenik, L. Ilkovics, D. Klemova, M. Kravec, F. Hubatka, Z. Dave, A. Hampl, V. Bryja, P. Matula and V. Pospichalova, J Extracell Vesicles 8, 1560808 (2019) doi: 10.1080/20013078.2018.1560808

7 K. Stejskal, D. Potesil and Z. Zdrahal, J Proteome Res 12, 3057-3062 (2013) doi: 10.1021/pr400183v

8 V. Demichev, C.B. Messner, S.I. Vernardis, K.S. Lilley and M. Ralser, Nat Methods 17, 41-44 (2020) doi: 10.1038/s41592-019-0638-x

9 G.H. Laurens van der Maaten, Journal of Machine Learning Research

9, 2579-2605 (2008)

10 E. Becht, L. McInnes, J. Healy, C.A. Dutertre, I.W.H. Kwok, L.G. Ng, F. Ginhoux and E.W. Newell, Nat Biotechnol, (2018) doi: 10.1038/nbt.4314

nbt.4314 [pii]

11 S. Van Gassen, B. Callebaut, M.J. Van Helden, B.N. Lambrecht, P. Demeester, T. Dhaene and Y. Saeys, Cytometry A 87, 636-645 (2015) doi: 10.1002/cyto.a.22625

12 J.H. Levine, E.F. Simonds, S.C. Bendall, K.L. Davis, A.D. Amir el, M.D. Tadmor, O. Litvin, H.G. Fienberg, A. Jager, E.R. Zunder, R. Finck, A.L. Gedman, I. Radtke, J.R. Downing, D. Pe'er and G.P. Nolan, Cell 162, 184-197 (2015) doi: 10.1016/j.cell.2015.05.047

S0092-8674(15)00637-6 [pii]

13 N. Samusik, Z. Good, M.H. Spitzer, K.L. Davis and G.P. Nolan, Nat Methods 13, 493-496 (2016) doi: 10.1038/nmeth.3863

nmeth.3863 [pii]

**Supplementary Figure Legends**

**Supplementary Figure S1. High-throughput profiling of surface molecules deregulated in docetaxel-resistant PCa models *in vitro*.** (A) The workflow scheme depicting a complete gating strategy (removal of dead cells, cell aggregates, and debris) is used to analyze all flow cytometric data in this manuscript, and the deconvolution of fluorescent barcoding is utilized for the high-throughput screen. Detailed information is provided in Supplementary Materials and Methods. (B) A heatmap shows the median fluorescence index (MFI) for all models and all antigens that were identified on the surface of at least one cell line. (C) The expression profile of antigens robustly changed in both DU145 and PC3 docetaxel-resistant models compared to their docetaxel-sensitive counterparts. The Y-axis indicates the median fluorescence index (median fluorescence intensity of the specific antigen normalized to the median fluorescence intensity of ISO control).

**Supplementary Figure S2. Profiling of extracellular vesicles from docetaxel-resistant cells.** (A) Representative cryo-EM images of EV samples, close-up at the top and overview at the bottom. (B) DLS measurements of isolated EVs. The graph shows the average of 3 separate measurements for each sample. (C) WB analyses of EVs markers (HSP70, FLOT1, CD9) and negative markers (golgin-97 and Ac-α-tubulin). *indicates overlay in an empty well. (D) Heatmap displaying expression profile of 12 molecules selected from initial flow cytometry screen in docetaxel-resistant vs. docetaxel-sensitive DU145 and PC3 cells (n=2). (E) Venn diagram overlay of EV proteins upregulated in docetaxel-resistant DU145 and PC3 cells as compared to their control relatives.

**Supplementary Figure S3. Validation of *in vitro* findings using PCa patient-derived xenografts.** Tumors derived from PDXs were collected and dissociated, and single-cell suspensions were stained as described in Materials and Methods. The plot displays the % positivity (x-axis) of particular antigens in PDX models. Data represent mean ± SEM from four independent tumors. ***, P<0.0001; **, P<0.01; *, P<0.05, ns, not significant by unpaired *t*-test.

**Supplementary Figure S4. Description of the clusters and patient sample distribution within the data from dissociated primary prostate tumor samples.** (A) The bar plot shows the number of cells within each FlowSOM population. (B) The lines indicate the expression profile of the six most deregulated antigens in "sensitive" population 1 (blue) and "resistant" population 3 (red) determined by FlowSOM in prostate cancer patient samples.

**Supplementary Figure S5. Multiple clustering and visualization analysis of the data from dissociated primary prostate tumor samples.** The plots represent the clustering and visualization of clusters using different algorithms. UMAP (A), tSNE (B), and TriMap (C) algorithms were utilized for dimension reduction and map creation. To verify the presence of a "resistant" population/cluster, three different clustering algorithms were applied: FlowSOM (related to the data in Figure 3B and 3C), X-shift (corresponding heatmap in D), and PhenoGraph (corresponding heatmap in E). All combinations of visualization and clustering algorithms resulted in identifying the desired population manifesting the same expression profile based on the six most deregulated surface antigens (FlowSOM - pop 3; X-shift - cluster 4 and PhenoGraph - cluster 9).

**Supplementary Figure S6. Formalin fixation is not compatible with the SSEA4 staining.** Cells with the known expression of SSEA4 were grown on glass coverslips and frozen at -80 °C. After thawing and formalin fixation for ten minutes (the intended procedure for tissue cryosections), the immunofluorescence staining was performed with the antibody against SSEA4 (clone MC13-70), as described in Methods. This pretreatment led to unspecific signals in SK-BR-3 cells (C; SSEA4 negative), which cannot be distinguished from MDA-MB-231 cells (A, SSEA4 positive). No signal was found with the omission of the primary antibody in either cell line (B and D).

**Supplementary Figure S7. Correlation of CD95 and SSEA-4 expression and disease prognosis.** (A) The scheme illustrates the precursors and enzymes implicated in the anabolism of SSEA-4, together with the consequences of its overexpression. (B) Expression of EpCAM, CD9, CD44 and CD59 in patient samples pre- or post-docetaxel-based therapy (n=11). The PC3 ctrl and PC3 DOC cells were seeded onto 96 well plates in a standard density of 20,000 cells/cm^2^ and treated with an increasing dose of CD95L and its enhancer after 24 hours in technical multiplicate (n=5) for 72 hours. The plotted data show normalized mean fluorescence values obtained after detection using CyQuant Cell Proliferation Assay ± S.D. from three independent repetitions. *indicates p<0.05 (t-test). (D, E) Correlation analysis of ST3GAL2 and z-score of the genes implicated in the resistance to antimicrotubule agents (Biocarta Pathway - Harmonizome) using all available data from TCGA samples (n=301) (D) and data from high-grade (GS≥8) prostate cancer patients (n=75) (E). (F-H) Correlation analysis of (F) ST3GAL2 and CD95 and (G) CD95 or (H) SSEA-4 and surface antigens upregulated in likely DOC sensitive subpopulation (Pop1; in blue) or upregulated in likely DOC resistant subpopulation, (Pop3; in red), according to the PDX fingerprints. Data portray the expression of antigens in patients suffering from high-grade (GS<7) prostate cancer disease (n=88) (TCGA).

**Supplementary Tables**

**Table S1.** LegendScreen - % of positivity.

**Table S2.** LegendScreen - MFI of 332 surface molecules (normalized to ISO).

**Table S3.** Overview of prostate cancer patient specimens.

**Table S4.** Overview of antibodies and reagents used for (spectral) flow cytometry.
