# Supplementary material for: Pre-existing cell subpopulations in primary prostate cancer tumors display surface fingerprints of docetaxel-resistant cells
Source: Cell Oncol (Dordr). 2024 Aug 20;48(1):205–18. doi: 10.1007/s13402-024-00982-2 (PMC11850551; doi:10.1007/s13402-024-00982-2)
Supplement: Supplementary file 3 — Supplementary Material 3 [file 13402_2024_982_MOESM3_ESM.pdf]

Supplementary Table 1. LegendScreen - % of positivity.

	MARKER	DU145	DU145 DOC	PC3	PC3 DOC
1	CD1a	1,6	1,8	1	2,2
2	CD1b	1,1	1,6	0,2	1
3	CD1c	1,1	0,9	0,1	1
4	CD1d	0,7	0,7	0	1,9
5	CD2	1,1	0,9	0,1	0,9
6	CD3	0,7	0,7	0,3	1,4
7	CD4	1,3	1,2	0,2	0,7
8	CD5	1,2	1,9	0,2	1,1
9	CD6	1,5	1,9	0,3	1,3
10	CD7	1,6	0,7	0,3	1,9
11	CD8a	1,7	1,9	0,1	1,3
12	CD9	87,8	97,9	67,4	92,3
13	CD10	1,5	1,9	0,8	3,2
14	CD11a	0,9	0,8	0,1	0,7
15	CD11b	0,9	0,8	0,1	1,1
16	CD11b	1,3	0,9	0,2	1
17	CD11c	1,4	1,4	0,2	0,9
18	CD13	2,4	2,6	36,7	82
19	CD14	0,6	0,8	0,7	2,8
20	CD15	34	2,3	0,2	1,9
21	CD16	2,2	1,9	0,1	1,4
22	CD18	2,8	2,2	0,3	2
23	CD19	3,6	4,5	0,6	2,6
24	CD20	0,7	0,6	0,2	1,5
25	CD21	1,5	0,8	0,4	21,4
26	CD22	1,1	1	0,1	0,6
27	CD23	0,9	0,7	0,2	0,5
28	CD24	31,3	15,5	39,6	74,4
29	CD25	1,2	1,1	0,3	1,1
30	CD26	1,2	1	2,7	57,2
31	CD27	1,1	1	0,3	1,1
32	CD28	1,5	1,5	0,1	0,7
33	CD29	99,2	99,2	98,5	99,1
34	CD30	2,8	2	0,7	2
35	CD31	3	3,8	0,3	2,6
36	CD32	0,5	0,4	0,2	0,9
37	CD33	0,7	0,6	0,1	1
38	CD34	0,8	0,9	0,1	0,6
39	CD35	1,2	0,5	0	0,8
40	CD36	1,1	0,5	0,2	2,4
41	CD38	15,4	6,2	0,1	1
42	CD39	1,7	1,4	0,2	1,6
43	CD40	3,8	2,4	0,5	6,6
44	CD41	3,3	2,4	0,2	2,4
45	CD42b	4,1	3,7	0,2	1,6
46	CD43	48,8	18	0,6	4,6
47	CD44	66,6	99,3	97,3	99,9
48	CD45	0,9	1	0,1	0,7
49	CD45RA	0,5	0,3	0,1	1,1

50	CD45RB	0,3	0,4	0,1	0,8
51	CD45RO	0,5	0,6	0,1	1,2
52	CD46	63,9	13,6	0,9	12,6
53	CD47	98,4	96,9	96,6	97,3
54	CD48	5,3	3,1	0,7	2
55	CD49a	10,7	6,8	0,7	2,5
56	CD49c	99,5	99,7	95	99,3
57	CD49d	48,8	31,3	3,6	21,9
58	CD49e	96,2	75,5	19,5	44,1
59	CD49f	84,4	51	87,6	95,3
60	CD50	1	0,9	0,2	0,8
61	CD51	55,8	32,6	13,8	13,4
62	CD51,CD61	1,4	1,4	0,5	1,6
63	CD52	0,7	0,9	0,3	2,4
64	CD53	0,8	1,3	0,3	0,8
65	CD54	99,8	99,7	41,6	89,5
66	CD55	99,9	96,7	98,9	100
67	CD56	41	1,7	2,4	18,6
68	CD57	2	0,9	1,9	31,5
69	CD58	98,6	97,7	91	98,6
70	CD59	98,4	97,4	97	96,6
71	CD61	2,5	2,6	0,9	2,9
72	CD62E	0,8	0,9	0,1	0,9
73	CD62L	0,9	0,9	0,1	0,9
74	CD62P	0,9	0,9	0,2	0,5
75	CD63	67,4	92,6	84,7	95,6
76	CD64	1,4	1,4	0,4	1,6
77	CD66a/c/e	0,8	0,8	5,6	9,1
78	CD66b	0,7	0,4	0,5	0,9
79	CD69	0,8	1,1	0,6	1,3
80	CD70	67,4	98	53,5	91,6
81	CD71	96,6	96,4	99,3	99,9
82	CD73	96,2	90,5	26,4	70,4
83	CD74	8,2	4,7	1,4	10,1
84	CD79b	1,1	1,6	0,3	1,7
85	CD80	0,9	1,2	0,1	1,2
86	CD81	96,6	98,1	90,9	97,4
87	CD82	6,3	6,7	1,8	10,1
88	CD83	1,4	2,1	0,5	2
89	CD84	0,9	0,8	0,5	1,3
90	CD85	0,6	0,6	0,2	0,6
91	CD85d	1	1,1	0,4	0,9
92	CD85	1,7	1,8	2,9	2,1
93	CD85h	1,3	1,6	1,5	9,8
94	CD85	0,7	1,2	1,6	2
95	CD85k	1,2	1,2	0,4	1,1
96	CD86	0,9	1,1	0,1	1,1
97	CD87	2,8	3	0,4	2,2
98	CD88	1,3	1,5	0,2	1,6
99	CD89	2,6	3,1	0,2	2,5
100	CD90	3	2,8	0,3	2,8
101	CD93	5,1	5	0,5	3,8

102	CD94	3,6	3,4	0,2	2,3
103	CD95	34,4	88,2	1	43
104	CD96	2	2,9	0,1	1,5
105	CD97	78,7	84,6	50,2	95,7
106	CD99	98,8	98,9	98,5	99,1
107	CD100	1,9	2	0,3	1,3
108	CD101	1,5	2	0,3	0,6
109	CD102	1,2	1,3	0,1	2,1
110	CD103	2	1,8	0,3	1,4
111	CD104	2,1	1,6	31,6	37,2
112	CD105	2,6	2,9	0,4	2,1
113	CD106	3,5	2,4	0,2	1,8
114	CD107a	11,8	20,2	2	21
115	CD108	5,5	2,3	7,9	43,2
116	CD109	1,8	2,3	39,5	76,4
117	CD111	12,8	42,6	0,5	20,2
118	CD112	94,9	93,7	20,3	93,5
119	CD114	1,4	1,3	0,1	0,9
120	CD115	1	0,8	0,4	0,7
121	CD116	2,6	2,5	0,3	1,6
122	CD117	1,6	1,5	0,1	0,8
123	CD119	4,6	3,3	0,5	4
124	CD122	2	1,4	0,2	1,2
125	CD123	1,9	1,7	0,4	1,2
126	CD124	1,6	1,3	0,8	3,3
127	CD126	1,9	1,2	0,1	1,6
128	CD127	2	2,1	0,4	9,3
129	CD129	1,1	1,5	0,6	2,7
130	CD131	1,9	2,1	0,1	1,3
131	CD132	0,5	0,8	0,4	0,7
132	CD134	1,6	1,9	0,2	0,9
133	CD135	1,9	1,7	0,1	1
134	CD137	1,8	1,8	0,1	1,3
135	4-1BB Ligand	3,2	3,3	0,3	1,7
136	CD138	5,3	3,7	0,5	7,8
137	CD140a	2,8	2,3	0,2	2,1
138	CD140b	2,4	2,5	0,3	2,4
139	CD141	8,6	2,8	3,3	2,6
140	CD143	2,8	2,1	0,5	4
141	CD144	1,1	1,4	0,4	2,9
142	CD146	91,4	93,4	34,4	87,8
143	CD148	3	1,8	0,4	1,3
144	CD150	1,3	1,4	0,1	0,9
145	CD152	1,2	1	0,2	0,9
146	CD154	1,9	1,3	0,3	1
147	CD155	100	99,8	88	99,4
148	CD156c	98,4	97,2	86,8	98,4
149	CD158	1,9	1,5	0,5	4,4
150	CD158b	1,2	1	0,3	2,3
151	CD158d	4,6	3,5	0,4	3,1
152	CD158e1	1,8	1,6	0,1	1,3
153	CD158f	2,2	2,3	0,3	1,9

154	CD161	2,9	2,7	0,1	1,2
155	CD162	1,7	1,5	0,2	0,7
156	CD163	2,3	1,7	0,4	1,9
157	CD164	65,5	63,3	60	97,9
158	CD165	80	43,4	13,4	84,3
159	CD166	73,7	99,8	98,4	99,8
160	CD167a	7,4	3,9	2,2	2,5
161	CD169	4,2	2,3	0,7	2,3
162	CD170	34,2	16,3	63,6	89,8
163	CD172a	11,9	13,6	18,3	85,6
164	CD172b	1,2	1	0,3	1,8
165	CD172g	2	2	0,4	1,6
166	CD178	2,6	1,9	0,3	2
167	CD179a	1,7	1,9	0,3	1,1
168	CD179b	1,2	1,2	0,2	0,9
169	CD180	1,2	1,1	0,1	1,1
170	CD181	1	1,2	0,4	1,4
171	CD182	1,3	1,5	0,4	1,8
172	CD183	3,4	2,5	0,4	3,4
173	CD184	1,8	1,5	0,6	4,1
174	CD193	0,9	1	0,5	2,1
175	CD195	5,1	5,8	3,4	5,7
176	CD196	1,1	1,3	0,5	3,7
177	CD197	0,9	1,7	0,8	1,8
178	CD200	1,5	1,2	0,4	0,8
179	CD200R	5,4	4,8	0,7	2,8
180	CD201	13,7	64,3	23,3	86,2
181	CD202b	1,6	1,8	0,5	1,7
182	CD203c	1,4	1,7	0,7	2,1
183	CD205	1,2	1,7	0,3	1,6
184	CD206	1,5	1,5	0,5	1,2
185	CD207	1,4	1,5	0,2	1,4
186	CD209	0,7	0,8	0,5	2,1
187	CD210	1	1	0,8	1,6
188	CD213α2	1,2	2,2	1,1	56,3
189	CD215	1	1,7	0,5	14,1
190	CD218a	2	1,6	0,4	4,3
191	CD220	3,3	1	1,9	35,5
192	CD221	17,3	6,7	0,5	5
193	CD226	6,3	4,1	0,3	3,5
194	CD229	6	4,4	0,3	3,9
195	CD231	3,2	3,6	0,5	3
196	CD235ab	3,6	3,3	1,7	4,8
197	CD243	2,7	54,5	0,7	60,5
198	CD244	3,9	3,6	0,1	3
199	CD245	11,7	10,4	3,6	20,2
200	CD252	3,1	3,7	0,3	9
201	CD253	3,1	3,7	0,2	1,8
202	CD254	4,3	5,5	0,6	4,3
203	CD255	1,5	2,1	0,3	1,2
204	CD257	12,1	3,7	1	24,4
205	CD258	1,5	1,3	0,2	2,1

206	CD261	9,2	5,4	0,5	6,1
207	CD262	89,3	44,8	6,7	39,7
208	CD263	4,2	4,6	0,3	3,3
209	CD266	41,4	37,2	1,3	27,8
210	CD267	1,7	1,8	0,9	1,6
211	CD268	4,5	3,4	0,4	3,8
212	HVEM	3,4	3,2	0,3	3,6
213	CD271	6,1	6	0,5	5,4
214	CD273	0,8	1,1	0,3	1,3
215	CD274	3,4	11,1	14,9	20,1
216	CD275	4,4	3,1	0,7	17,7
217	CD276	89,7	96	69,8	95
218	CD277	10,6	8,6	0,4	5,3
219	CD278	1,5	1,1	1	3,4
220	CD279	5,9	4,6	0,7	4,9
221	CD282	2,7	1,9	0,8	6,2
222	CD284	5,1	5	2,8	13,4
223	CD286	4,5	3,3	0,2	3,1
224	CD290	3,6	3	0,3	2,4
225	CD294	3,3	2,7	1,1	2,5
226	CD298	98,5	97,8	96,2	97,4
227	CD300e	1,1	0,7	0,8	0,7
228	CD300F	2,1	1,9	0,5	1,8
229	CD301	3,8	2,7	2,1	9,1
230	CD303	2,6	1,7	1,1	5
231	CD304	7,5	3,1	65,4	94,8
232	CD307e	2,7	1,7	0,9	5,2
233	FcRL4	3,8	2,3	2,2	7,4
234	CD314	7,2	5,4	0,7	5,9
235	CD317	19	8,6	2,8	12,3
236	CD318	98,9	95,5	95,7	99,7
237	CD319	7,6	5,6	2	13,7
238	CD324	40,3	2,5	4,9	15
239	CD325	1,3	1,4	0,4	1,5
240	CD326	98,7	67,4	95,7	98,8
241	CD328	7,7	5,7	2,1	6,5
242	CD334	10,1	6,6	0,5	7,4
243	CD335	6,4	3,9	1	5,8
244	CD336	8,2	6,2	0,9	5,5
245	CD337	4,7	3,2	0,7	3,8
246	CD338	8,9	6,2	1,2	8,3
247	CD340	41,9	32,6	2,9	42,5
248	CD344	8,1	4,5	0,6	13,4
249	CD351	13,3	8,5	0,6	8,1
250	CD352	0,9	1,4	0,1	1
251	CD354	1,1	0,9	0,1	1
252	CD355	1,2	1	1,1	2,2
253	CD357	6,5	8,4	1,2	5,4
254	CD360	14,3	9,2	2,4	10,3
255	β2-microglobu	99	98,4	97,9	98,9
256	CD272	2,6	1,7	2,7	7
257	C3aR	3,1	2,8	2,1	10,1

258	C5L2	2,6	2,2	3,5	7,6
259	CCR10	2,4	1,4	2,1	6,7
260	CLEC12A	3	2,6	1,8	7
261	CLEC9A	3	2,6	1,6	5,4
262	CX3CR1	0,8	0,9	1,9	1,4
263	CXCR7	2,4	4,1	1,5	4,1
264	Delta Opioid F	1,6	1,9	1,3	3
265	DLL1	11	5,5	1,6	7,7
266	DLL4	13,4	7,6	1,6	9,5
267	DR3	8,9	6,2	2,9	8,2
268	EGFR	97,6	94,3	52,6	94,2
269	erbB3	16,7	9,2	6,7	20,6
270	FcεRIα	1,7	1,5	4,5	7,1
271	FcRL6	2,2	2	3,3	6
272	Galectin-9	3,9	3,3	0,6	2,5
273	GARP	2,4	1,7	2,4	5,3
274	HLA-A,B,C	92,3	94	85,4	95,4
275	HLA-A2	0,7	0,6	1,5	1,2
276	HLA-DQ	2,7	1,6	1,1	2,6
277	HLA-DR	3	2,1	3,9	9,3
278	HLA-E	13,8	8	5,3	11,9
279	HLA-G	1,7	1,4	5,3	6,5
280	IFN-γ R b chai	1,7	1,8	1,8	4,9
281	Ig light chain κ	4,6	3,2	0,9	3,2
282	Ig light chain λ	1,3	0,9	0,1	3
283	IgD	1,7	1,8	0,8	4,5
284	IgM	4,5	3,2	0,9	3,2
285	IL-28RA	1,1	0,7	0,8	1,8
286	Integrin α9β1	2,8	2,7	0,4	2,1
287	integrin β5	48,3	45,9	2,7	6,1
288	Integrin β7	2,1	1,8	0,9	0,8
289	Jagged 2	4,4	4,2	0,7	3,2
290	LAP	3,8	3,9	1,2	2,5
291	Lymphotoxin β	7,8	10,7	5,5	40,2
292	Mac-2	3,5	3,7	0,1	3,8
293	MAIR-II	4,9	5,5	0,4	4,5
294	MICA/MICB	42	10,2	1,6	7,1
295	SUSD2	5,6	3,6	0,4	6,3
296	SUSD2	10,2	6	0,4	5,1
297	MSC	91	87	11,4	76,5
298	MSC,NPC	11,2	6	3,7	50,1
299	TNAP	2,8	3,2	0,3	2,9
300	NKp80	7,7	3,9	0,7	4,1
301	Notch 1	3,7	3,4	1,6	4,6
302	Notch 2	2,4	2,7	2,4	19
303	Notch3	4,7	3,8	0,3	3,9
304	Notch 4	2,6	2	0,2	4,9
305	NPC	6,7	5,4	0,3	4,9
306	Podoplanin	3,7	2,1	7,1	7,9
307	Pre-BCR	5,7	5,1	1,1	5
308	PSMA	5,1	5,2	1,1	4,1
309	Siglec-10	3,4	3,8	0,2	2

310	Siglec-8	2,8	3,3	0,9	3
311	Siglec-9	3,6	3	0,6	2,6
312	SSEA-1	10,3	1,1	1,9	2
313	SSEA-3	1,5	1,6	0,5	4,1
314	SSEA-4	20,4	73,9	85	95,9
315	SSEA-5	47,2	48,5	72,2	89,6
316	TCR γ/δ	6,9	6,8	3,3	7,1
317	TCR Vβ13.2	7,6	7,1	1,6	7,5
318	TCR Vβ23	5,5	4,7	0,9	4,4
319	TCR Vβ8	4	3,6	2,5	7,3
320	TCR Vβ9	2,4	2,3	1,9	6,1
321	TCR Vδ2	2,3	2,6	0,8	1,6
322	Vγ9	3,5	3,7	0,4	2,5
323	TCR Vα24-Jα'	2,1	2,1	0,4	1,8
324	TCR Vα7.2	4,2	3,3	0,5	3,4
325	TCR α/β	4,1	3,4	1,8	3,5
326	Tim-1	3	2,4	0,6	1,5
327	Tim-3	5,4	4,3	0,4	2,9
328	Tim-4	7,7	5,1	0,3	4
329	TLT-2	6,5	5,1	4,5	5,7
330	TRA-1-60-R	9	21,8	5,4	10,9
331	TRA-1-81	3,4	4,5	2,7	3,4
332	TSLPR	3,8	3,8	0,6	2,3

Supplementary Table 2. LegendScreen - MFI of 332 surface molecules (normalized to ISO).

	MARKER	DU145	DU145 DOC	PC3	PC3 DOC
1	CD1a	0,726	0,730	0,573	0,706
2	CD1b	0,681	0,669	0,562	0,630
3	CD1c	0,704	0,720	0,539	0,649
4	CD1d	0,715	0,715	0,637	0,715
5	CD2	0,676	0,671	0,633	0,691
6	CD3	0,687	0,684	0,553	0,668
7	CD4	0,745	0,712	0,650	0,748
8	CD5	0,701	0,712	0,552	0,672
9	CD6	0,781	0,789	0,777	0,821
10	CD7	0,837	0,804	0,738	0,816
11	CD8a	0,823	0,815	0,702	0,804
12	CD9	4,499	7,882	2,551	4,032
13	CD10	0,792	0,789	0,755	0,856
14	CD11a	0,681	0,663	0,593	0,635
15	CD11b	0,726	0,704	0,610	0,710
16	CD11b	0,745	0,740	0,668	0,753
17	CD11c	0,759	0,733	0,655	0,748
18	CD13	0,834	0,884	1,627	4,820
19	CD14	0,740	0,751	0,731	0,850
20	CD15	1,737	0,907	0,756	0,875
21	CD16	0,859	0,861	0,785	0,861
22	CD18	0,848	0,863	0,759	0,901
23	CD19	0,970	1,030	0,774	0,939
24	CD20	0,789	0,820	0,664	0,765
25	CD21	0,892	0,781	0,824	1,430
26	CD22	0,751	0,743	0,667	0,745
27	CD23	0,767	0,725	0,674	0,717
28	CD24	1,429	1,178	1,611	2,635
29	CD25	0,806	0,774	0,742	0,807
30	CD26	0,911	0,909	0,919	2,043
31	CD27	0,756	0,735	0,737	0,751
32	CD28	0,859	0,881	0,567	0,734
33	CD29	50,535	38,768	12,006	24,731
34	CD30	0,953	0,922	0,809	0,904
35	CD31	1,061	1,145	0,781	0,981
36	CD32	0,801	0,789	0,624	0,731
37	CD33	0,756	0,735	0,621	0,719
38	CD34	0,740	0,722	0,647	0,715
39	CD35	0,759	0,722	0,657	0,727
40	CD36	0,878	0,863	0,750	0,857
41	CD38	1,249	1,119	0,735	0,823
42	CD39	0,856	0,848	0,755	0,880
43	CD40	1,042	0,948	0,885	1,236
44	CD41	1,072	1,012	0,778	0,942
45	CD42b	1,066	1,037	0,829	0,984
46	CD43	2,224	1,355	0,876	1,064
47	CD44	5,803	36,081	6,050	28,431

48	CD45	0,806	0,789	0,667	0,753
49	CD45RA	0,756	0,745	0,663	0,746
50	CD45RB	0,784	0,812	0,662	0,751
51	CD45RO	0,823	0,799	0,722	0,800
52	CD46	2,612	1,545	0,967	1,394
53	CD47	6,925	6,962	4,971	7,522
54	CD48	1,086	1,076	0,888	0,894
55	CD49a	1,274	1,186	0,858	0,927
56	CD49c	22,235	22,029	6,214	12,810
57	CD49d	2,249	1,957	1,188	1,548
58	CD49e	7,396	4,183	1,411	1,777
59	CD49f	4,751	2,866	3,177	4,347
60	CD50	0,828	0,873	0,737	0,817
61	CD51	2,986	2,213	1,333	1,272
62	CD51,CD61	0,853	0,881	0,798	0,843
63	CD52	0,828	0,897	0,777	0,875
64	CD53	0,806	0,784	0,762	0,812
65	CD54	22,584	17,702	1,865	3,643
66	CD55	11,263	5,266	5,660	15,434
67	CD56	2,008	0,920	1,030	1,355
68	CD57	1,083	1,099	0,970	1,526
69	CD58	6,765	6,066	3,054	5,062
70	CD59	17,474	35,748	11,593	17,076
71	CD61	1,006	1,037	0,870	0,979
72	CD62E	0,792	0,774	0,771	0,772
73	CD62L	0,789	0,771	0,702	0,736
74	CD62P	0,773	0,802	0,705	0,758
75	CD63	2,950	8,922	3,594	6,106
76	CD64	0,812	0,804	0,814	0,857
77	CD66a/c/e	0,834	0,802	0,907	0,904
78	CD66b	0,889	0,809	0,805	0,856
79	CD69	0,853	0,832	0,878	0,866
80	CD70	2,853	7,182	2,131	3,643
81	CD71	4,066	10,786	11,867	27,582
82	CD73	11,307	5,256	1,518	2,456
83	CD74	1,338	1,173	1,008	1,279
84	CD79b	0,812	0,789	0,701	0,758
85	CD80	0,756	0,745	0,627	0,706
86	CD81	7,255	12,664	4,005	8,473
87	CD82	1,166	1,155	0,880	1,310
88	CD83	0,864	0,799	0,881	0,885
89	CD84	0,825	0,799	0,783	0,817
90	CD85	0,770	0,751	0,728	0,764
91	CD85d	0,801	0,774	0,781	0,804
92	CD85	0,961	1,002	1,098	1,059
93	CD85h	1,047	1,068	0,977	1,192
94	CD85	0,878	0,897	0,855	0,896
95	CD85k	0,859	0,868	0,778	0,866
96	CD86	0,825	0,799	0,633	0,748
97	CD87	0,828	0,799	0,650	0,731

98	CD88	0,806	0,781	0,680	0,736
99	CD89	0,853	0,848	0,682	0,805
100	CD90	0,856	0,848	0,843	0,901
101	CD93	0,939	0,973	0,789	0,944
102	CD94	0,911	0,930	0,838	0,944
103	CD95	1,911	4,485	0,971	1,867
104	CD96	0,817	0,871	0,617	0,765
105	CD97	3,305	4,826	2,037	4,009
106	CD99	28,842	38,409	14,215	18,193
107	CD100	0,853	0,799	0,624	0,833
108	CD101	0,778	0,774	0,647	0,673
109	CD102	0,837	0,812	0,636	0,724
110	CD103	0,823	0,799	0,755	0,800
111	CD104	0,992	0,889	1,490	1,637
112	CD105	0,864	0,871	0,809	0,899
113	CD106	0,909	0,884	0,699	0,812
114	CD107a	1,316	1,542	0,939	1,449
115	CD108	1,102	1,137	1,067	1,980
116	CD109	0,828	0,879	1,851	2,777
117	CD111	1,404	2,213	0,798	1,550
118	CD112	4,842	4,657	1,675	3,162
119	CD114	0,795	0,761	0,592	0,686
120	CD115	0,795	0,784	0,778	0,823
121	CD116	0,900	0,858	0,755	0,857
122	CD117	0,812	0,774	0,702	0,765
123	CD119	1,086	1,030	0,816	1,142
124	CD122	0,795	0,781	0,783	0,771
125	CD123	0,864	0,840	0,851	0,889
126	CD124	0,931	0,891	0,886	0,942
127	CD126	0,878	0,832	0,748	0,857
128	CD127	0,859	0,850	0,777	1,076
129	CD129	0,994	1,173	0,773	0,942
130	CD131	0,917	0,907	0,653	0,807
131	CD132	0,781	0,774	0,633	0,729
132	CD134	0,825	0,884	0,708	0,746
133	CD135	0,823	0,820	0,652	0,757
134	CD137	0,837	0,825	0,721	0,772
135	4-1BB Ligand	1,083	0,986	0,674	0,852
136	CD138	1,188	1,055	0,924	1,227
137	CD140a	0,945	0,938	0,840	0,939
138	CD140b	0,928	0,891	0,781	0,911
139	CD141	1,114	0,948	0,951	0,875
140	CD143	0,922	0,920	0,813	1,007
141	CD144	0,942	0,955	0,794	0,885
142	CD146	12,997	11,455	1,590	3,494
143	CD148	1,102	1,002	0,752	0,932
144	CD150	0,834	0,822	0,738	0,805
145	CD152	0,812	0,794	0,769	0,817
146	CD154	0,842	0,812	0,817	0,816
147	CD155	17,740	12,018	3,183	6,106

148	CD156c	6,042	5,694	2,841	4,571
149	CD158	0,942	0,897	0,774	0,890
150	CD158b	0,953	0,909	0,759	0,885
151	CD158d	1,006	0,986	0,871	0,960
152	CD158e1	0,848	0,835	0,735	0,823
153	CD158f	0,917	0,930	0,778	0,878
154	CD161	0,961	0,976	0,637	0,783
155	CD162	0,845	0,822	0,589	0,692
156	CD163	0,956	0,930	0,893	0,927
157	CD164	2,831	2,997	2,321	7,338
158	CD165	3,305	2,275	1,424	2,916
159	CD166	9,305	16,158	6,312	13,358
160	CD167a	1,238	0,994	0,860	1,024
161	CD169	1,028	0,968	0,881	0,975
162	CD170	1,850	1,460	2,470	4,726
163	CD172a	1,382	1,606	1,581	3,120
164	CD172b	0,823	0,812	0,682	0,781
165	CD172g	0,934	0,935	0,816	0,880
166	CD178	0,964	0,986	0,792	0,948
167	CD179a	0,892	0,871	0,695	0,731
168	CD179b	0,834	0,820	0,706	0,745
169	CD180	0,834	0,822	0,786	0,816
170	CD181	0,903	0,853	0,747	0,824
171	CD182	0,875	0,868	0,835	0,871
172	CD183	0,970	0,935	0,759	0,904
173	CD184	0,981	0,950	0,819	0,939
174	CD193	0,909	0,907	0,722	0,840
175	CD195	1,163	1,204	1,147	1,151
176	CD196	0,934	0,884	0,765	0,949
177	CD197	0,837	0,820	0,774	0,847
178	CD200	0,881	0,873	0,727	0,790
179	CD200R	1,183	1,214	0,714	0,880
180	CD201	1,612	3,348	1,432	4,110
181	CD202b	0,909	0,909	0,881	0,880
182	CD203c	0,942	0,912	0,880	0,927
183	CD205	0,898	0,884	0,837	0,885
184	CD206	0,878	0,850	0,876	0,861
185	CD207	0,903	0,909	0,794	0,863
186	CD209	0,889	0,871	0,781	0,852
187	CD210	0,939	0,909	0,798	0,918
188	CD213α2	0,825	0,871	0,792	2,565
189	CD215	0,856	0,840	0,659	0,963
190	CD218a	1,044	0,945	0,798	1,142
191	CD220	1,316	0,848	0,951	1,633
192	CD221	1,548	1,263	0,830	1,074
193	CD226	0,903	0,850	0,774	0,764
194	CD229	0,956	0,930	0,827	0,923
195	CD231	0,842	0,840	0,728	0,835
196	CD235ab	0,903	0,835	0,675	0,800
197	CD243	0,889	3,038	0,689	2,221

198	CD244	0,859	0,871	0,728	0,873
199	CD245	1,219	1,222	0,999	1,409
200	CD252	0,970	0,945	0,777	1,178
201	CD253	0,892	0,889	0,711	0,824
202	CD254	0,726	0,704	0,645	0,706
203	CD255	0,751	0,671	0,664	0,719
204	CD257	1,211	0,912	0,891	1,442
205	CD258	0,795	0,740	0,594	0,696
206	CD261	1,199	1,030	0,888	1,088
207	CD262	4,188	2,280	1,295	1,831
208	CD263	0,994	0,938	0,794	0,915
209	CD266	2,028	2,026	1,001	1,548
210	CD267	0,942	0,945	0,890	0,974
211	CD268	0,970	0,958	0,853	0,948
212	HVEM	0,997	0,981	0,866	1,088
213	CD271	1,122	1,127	0,883	1,074
214	CD273	0,698	0,671	0,750	0,804
215	CD274	1,072	1,463	1,231	1,335
216	CD275	1,053	1,068	0,964	1,506
217	CD276	4,133	5,884	2,349	3,629
218	CD277	1,288	1,304	0,926	1,099
219	CD278	0,997	0,938	0,911	0,968
220	CD279	1,083	1,040	0,973	1,090
221	CD282	1,033	1,048	0,935	1,076
222	CD284	1,172	1,153	1,026	1,173
223	CD286	1,061	1,027	0,935	1,041
224	CD290	1,044	1,009	0,917	0,981
225	CD294	1,175	1,171	0,975	1,099
226	CD298	24,690	26,547	7,126	18,568
227	CD300e	0,751	0,753	0,881	0,807
228	CD300F	0,886	0,881	0,868	0,911
229	CD301	1,180	1,148	0,999	1,168
230	CD303	1,114	1,050	0,916	1,066
231	CD304	1,429	1,281	2,149	4,828
232	CD307e	1,127	1,058	0,902	1,059
233	FcRL4	1,202	1,148	0,992	1,114
234	CD314	1,147	1,130	0,957	1,076
235	CD317	1,474	1,273	1,098	1,263
236	CD318	8,305	6,032	3,217	7,084
237	CD319	1,557	1,491	1,026	1,276
238	CD324	1,778	0,920	1,144	1,218
239	CD325	0,789	0,743	0,824	0,883
240	CD326	33,598	3,624	7,999	2,598
241	CD328	1,194	1,160	1,007	1,147
242	CD334	1,294	1,204	0,997	1,156
243	CD335	1,166	1,122	1,005	1,142
244	CD336	1,213	1,224	1,008	1,159
245	CD337	1,097	1,073	0,947	1,076
246	CD338	1,199	1,204	1,026	1,206
247	CD340	2,097	2,031	1,243	1,860

248	CD344	1,310	1,186	0,973	1,373
249	CD351	1,418	1,383	0,965	1,224
250	CD352	0,706	0,684	0,721	0,661
251	CD354	0,770	0,735	0,792	0,736
252	CD355	1,036	0,973	0,921	0,968
253	CD357	1,233	1,355	1,078	1,198
254	CD360	1,515	1,440	1,138	1,340
255	β2-microglobuli	70,355	47,615	19,344	36,291
256	CD272	1,180	1,163	1,030	1,170
257	C3aR	1,222	1,199	1,011	1,206
258	C5L2	1,241	1,191	1,067	1,178
259	CCR10	1,230	1,199	0,997	1,147
260	CLEC12A	1,302	1,314	0,999	1,175
261	CLEC9A	1,338	1,327	0,960	1,128
262	CX3CR1	0,770	0,722	0,774	0,748
263	CXCR7	0,825	0,781	0,773	0,804
264	Delta Opioid Re	1,022	1,030	0,840	0,974
265	DLL1	1,385	1,286	1,085	1,227
266	DLL4	1,404	1,386	1,045	1,281
267	DR3	1,385	1,327	1,125	1,289
268	EGFR	9,551	5,894	2,069	4,726
269	erbB3	1,961	1,773	1,134	1,446
270	FcεRIα	1,188	1,153	1,085	1,154
271	FcRL6	1,258	1,204	1,052	1,142
272	Galectin-9	1,194	1,186	0,914	1,088
273	GARP	1,341	1,299	1,005	1,121
274	HLA-A,B,C	7,075	6,747	2,854	5,340
275	HLA-A2	0,795	0,763	0,885	0,783
276	HLA-DQ	1,066	1,022	0,955	0,986
277	HLA-DR	1,374	1,329	1,106	1,236
278	HLA-E	1,488	1,463	1,260	1,381
279	HLA-G	1,199	1,160	1,078	1,133
280	IFN-γ R b chain	1,211	1,130	0,932	1,059
281	Ig light chain κ	1,219	1,163	0,977	1,076
282	Ig light chain λ	1,116	1,043	0,755	0,944
283	IgD	1,199	1,186	0,888	1,036
284	IgM	1,241	1,232	1,018	1,104
285	IL-28RA	1,091	1,048	0,876	0,958
286	Integrin α9β1	0,856	0,812	0,774	0,823
287	integrin β5	2,720	2,679	1,067	1,088
288	Integrin β7	0,825	0,804	0,847	0,833
289	Jagged 2	0,856	0,897	0,958	0,918
290	LAP	0,856	0,884	1,003	0,965
291	Lymphotoxin β	1,521	1,685	1,199	1,739
292	Mac-2	0,853	0,884	0,891	0,955
293	MAIR-II	0,909	0,899	0,822	0,883
294	MICA/MICB	2,438	1,578	0,993	1,041
295	SUSD2	1,036	0,994	0,824	0,932
296	SUSD2	0,997	0,955	0,801	0,922
297	MSC	6,623	4,728	1,421	2,829

298	MSC,NPC	1,133	0,994	1,071	1,996
299	TNAP	0,864	0,861	0,875	0,885
300	NKp80	1,075	0,986	0,984	1,012
301	Notch 1	0,864	0,884	1,030	1,041
302	Notch 2	1,083	1,140	1,037	1,440
303	Notch3	0,903	0,884	0,850	0,915
304	Notch 4	0,734	0,722	0,604	0,901
305	NPC	0,950	0,973	0,832	1,007
306	Podoplanin	1,014	0,991	1,055	1,093
307	Pre-BCR	1,011	1,066	0,988	1,007
308	PSMA	0,992	1,048	0,953	0,991
309	Siglec-10	0,953	1,012	0,842	0,932
310	Siglec-8	0,898	0,930	0,955	0,975
311	Siglec-9	0,931	0,935	0,937	0,963
312	SSEA-1	1,258	1,002	1,026	0,994
313	SSEA-3	0,903	0,920	1,090	1,076
314	SSEA-4	1,296	3,858	4,791	15,059
315	SSEA-5	2,130	2,346	5,917	6,555
316	TCR γ/δ	1,033	1,055	0,905	1,036
317	TCR Vβ13.2	1,066	1,109	1,039	1,142
318	TCR Vβ23	0,983	0,973	0,933	0,975
319	TCR Vβ8	1,141	1,163	0,970	1,071
320	TCR Vβ9	1,044	1,137	1,026	1,036
321	TCR Vδ2	0,956	1,009	0,964	0,949
322	Vγ9	0,917	0,996	0,886	0,918
323	TCR Vα24-Jα18	0,842	0,884	0,851	0,868
324	TCR Vα7.2	1,003	0,991	0,921	1,000
325	TCR α/β	1,006	1,043	1,060	1,047
326	Tim-1	0,970	0,958	0,916	0,942
327	Tim-3	1,025	1,032	0,878	0,974
328	Tim-4	1,055	1,055	0,860	0,975
329	TLT-2	1,064	1,101	1,164	1,126
330	TRA-1-60-R	1,277	1,732	1,151	1,276
331	TRA-1-81	1,125	1,273	1,026	1,095
332	TSLPR	1,055	1,066	0,944	1,012

Supplementary Table 3. Overview of prostate cancer patient specimens.

Sample No.	Sample ID	Diagnosis	Gleason score	Grade group	TNM	Ki67 (%)	iPSA	Therapy	Surgery	Last check	Follow-up	Docetaxel Tx
PCa1	2033	adenocarcinoma	4+3	3	pT3a, pN0, pMX	23	6,6	without adjuvant therapy	08.06.2020	2024, July 3	remission, without adjuvant therapy	no
PCa2	1981	adenocarcinoma	3+4	2	pT3a, pN0, pMX	2	4,77	without adjuvant therapy	13.12.2019	2024, July 3	remission, without adjuvant therapy	no
PCa3	2036	adenocarcinoma	4+3	3	pT3a, pN1, pMX	11	4,8	without adjuvant therapy	09.06.2020	2024, July 3	remission, without adjuvant therapy	no
PCa4	2046	adenocarcinoma	4+5	5	pT3b, pN1, pMX	21	15,44	hormonal therapy LHRH	23.06.2020	2023, January 4 - death due to pancreatic cancer	ADT since 2020, August 6; progression to metastatic CRPC and abiraterone acetate since 2022, April 21; inoperable pancreatic cancer was found 2022, May 3; and treated with chemotherapy regime FOLFINOX since 2022, August 31.	no
PCa5	2040	adenocarcinoma	4+5, GS 5 up to 5%	5	pT3a, pN0, pMX	17	16,2	without adjuvant therapy	15.06.2020	2021, November	remission, without adjuvant therapy	no
PCa6	2044	adenocarcinoma	4+3, GS 5 up to 5%	3	pT3a, pN0, pMX	8	5,32	without adjuvant therapy	23.06.2020	2024, March 21	remission, without adjuvant therapy	no
PCa7	2010	adenocarcinoma	4+3, GS 5 up to 5%	3	pT3a, pN1, pMX	9	5,13	without adjuvant therapy	18.02.2020	2024, March 14	ADT since 2020, November 23; without progression	no
PCa8	1591	adenocarcinoma	4+5	5	pT3b, pN0, pMX	4	9,94	salvage RT+ LHRH	31.01.2018	2024, July 3	salvage radiotherapy and ADT since 2020, September 24; without progression	no

Supplementary Table 4. Overview of antibodies and reagents used for (spectral) flow cytometry.

Antigen	Conjugate	Host	Isotype and clonality	Producer	Cat.No.	Final concentration
CD9	CF-Blue	Mouse	IgG2a,κ; clone VJ1/20	Immunostep	9CFB-100T	1:20
CD44	AF532	Rat	IgG2b; clone IM7	eBioscience	58-0441-82	1:40
CD59	PE	Mouse	IgG2a,κ; clone p282 (H19)	SONY	2123540	1:40
CD63	BV650	Mouse	IgG1,κ; clone H5C6	Biolegend	353026	1:20
CD70	BV786	Mouse	IgG3,κ; clone Ki-24	BD Horison	565338	1:20
CD71	VioGreen	Mouse	IgG1,κ; clone REA902	Miltenyi Biotec	130-115-035	1:20
CD81	PE-Cy7	Mouse	IgG1,κ; clone 5A6	SONY	2347560	1:40
CD95	PE-Cy5	Mouse	IgG1,κ; clone DX2	Biolegend	305610	1:320
CD97	BV605	Mouse	IgG1,κ; clone VIM3b	BD OptiBuild	742447	1:80
ALCAM/CD166	PerCP-Cy5.5	Mouse	IgG1,κ; clone 3A6	Biolegend	343908	1:80
CD201	BV711	Rat	IgG1,κ; clone RCR-252	BD OptiBuild	743555	1:80
SSEA-4	PE-CF594	Mouse	IgG3,κ; clone MC813-70	BD Horison	562487	1:20
EpCAM/CD326	BV421	Mouse	IgG2b,κ; clone 9C4	Biolegend	324220	1:200
hCD298	FITC	Recombinant Human	IgG1; clone REA217	Miltenyi Biotec	130-123-324	1:100
CD31	FITC	Mouse	IgG1,κ; clone WM59	SONY	2115520	1:100
CD45	FITC	Mouse	IgG1,κ; clone HI30	SONY	2120030	1:100
CD90	FITC	Mouse	IgG1,κ; clone Thy1	Biolegend	328108	1:100
**Viability**						
Live/Dead	Yellow			Invitrogen	L34959	1:500
	Green			Invitrogen	L23101	1:1000
**Barcoding**						
CellTrace	Violet			Invitrogen	C34557	1:500 - 1:10 000
	Far Red DDAO-SE			Invitrogen	C34553	1:1000 - 1:10 000
**Others**						
Super Bright Complete Staining Buffer				eBioscience	SB-4401-42	1:100
